# A cellulose-derived supramolecule for fast ion transport

**DOI:** 10.1126/sciadv.add2031

**Published:** 2022-12-09

**Authors:** Qi Dong, Xin Zhang, Ji Qian, Shuaiming He, Yimin Mao, Alexandra H. Brozena, Ye Zhang, Travis P. Pollard, Oleg A. Borodin, Yanbin Wang, Bhargav Sai Chava, Siddhartha Das, Peter Zavalij, Carlo U. Segre, Dongyang Zhu, Lin Xu, Yanliang Liang, Yan Yao, Robert M. Briber, Tian Li, Liangbing Hu

**Affiliations:** ^1^ Department of Materials Science and Engineering, University of Maryland College Park, College Park, MD 20742, USA.; ^2^ National Institute of Standards and Technology, Gaithersburg, MD 20783, USA.; ^3^ Department of Electrical and Computer Engineering, University of Houston, Houston, TX 77204, USA.; ^4^ Texas Center for Superconductivity at the University of Houston (TcSUH), Houston, TX 77204, USA.; ^5^ Battery Science Branch, Energy Science Division, Sensor and Electron Devices Directorate, DEVCOM Army Research Laboratory, Adelphi, MD 20783, USA.; ^6^ School of Mechanical Engineering, Purdue University, West Lafayette, IN 47907, USA.; ^7^ Department of Mechanical Engineering, University of Maryland College Park, College Park, MD 20742, USA.; ^8^ Department of Chemistry and Biochemistry, University of Maryland College Park, College Park, MD 20742, USA.; ^9^ Center for Synchrotron Radiation Research and Instrumentation (CSRRI), Illinois Institute of Technology, Physics Department, Chicago, IL 60616, USA.; ^10^ Center for Materials Innovation, University of Maryland College Park, College Park, MD 20742, USA.

## Abstract

Supramolecular frameworks have been widely synthesized for ion transport applications. However, conventional approaches of constructing ion transport pathways in supramolecular frameworks typically require complex processes and display poor scalability, high cost, and limited sustainability. Here, we report the scalable and cost-effective synthesis of an ion-conducting (e.g., Na^+^) cellulose-derived supramolecule (Na-CS) that features a three-dimensional, hierarchical, and crystalline structure composed of massively aligned, one-dimensional, and ångström-scale open channels. Using wood-based Na-CS as a model material, we achieve high ionic conductivities (e.g., 0.23 S/cm in 20 wt% NaOH at 25 °C) even with a highly dense microstructure, in stark contrast to conventional membranes that typically rely on large pores (e.g., submicrometers to a few micrometers) to obtain comparable ionic conductivities. This synthesis approach can be universally applied to a variety of cellulose materials beyond wood, including cotton textiles, fibers, paper, and ink, which suggests excellent potential for a number of applications such as ion-conductive membranes, ionic cables, and ionotronic devices.

## Teaser

A cellulose-derived supramolecule with aligned, one-dimensional and ångström-scale channels for fast ion transport is developed.

## INTRODUCTION

Recent development in ionic devices has spurred the discovery of a range of supramolecular frameworks, such as two-dimensional (2D) and 3D molecular ensembles as well as metal-organic frameworks (MOFs) (*1*, *2*). The highly ordered structure of these materials with their tailored ion transport pathways can incur unique ion transport behaviors such as improved ion selectivity, reduced resistance, surface charge-governed ion transport, and surface-mediated ion conduction, especially those with ångström-scale (subnanometers to a few nanometers) pores/channels (*3*–*6*). At this size regime, the solvation environment of the ions, ion pair configuration, ion-surface interactions, and ion motion can vastly differ from that in conventional porous materials with mesopores and macropores (*7*). As a result, substantial efforts have been dedicated to developing effective methods for synthesizing small (nanoscale and subnanoscale) pores/channels within supramolecular frameworks (*8*, *9*). However, these approaches are usually complex, expensive, and nonscalable, hindering the application of these promising materials for ion transport applications.

**Fig. 1.**
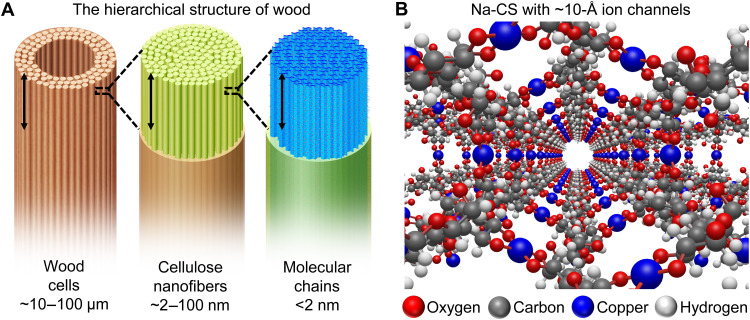
Molecular engineering of cellulose toward the highly ordered Na-CS. (**A**) Schematic diagrams of the hierarchical structure of cellulose in wood at different scales. The wood cells are typically in the range of ~10 to 100 μm in diameter (left), which contains cellulose nanofibers with diameters of ~2 to 100 nm (middle). The smallest unit of cellulose is composed of cellulose molecular chains at the subnanometer to nanometer scale (right). (**B**) Schematic image of the vastly aligned 1D channels formed by the cellulose molecular chains (ligands) and copper ions (metal nodes), which allow for fast ion transport. The structure is generated from crystal structure modeling based on XRD results. The channels have an available space of ~10 Å in diameter for ion conduction. The water molecules and Na^+^ ions are omitted in the drawing to clearly show the structure of the open channels.

Cellulose, which usually serves as an important structural component in the cell walls of wood and other plants, is the most abundant natural material on Earth. The sustainable and multiscale nature of cellulose, hierarchically composed of aligned fibers, nanofibers, and 1D molecular chains (Fig. 1A), makes cellulose a promising candidate for ionic device applications (*10*–*12*). A range of nanoscale engineering strategies have been found to use the small building blocks of cellulose nanofibers [also known as nanofibrillated cellulose or nanocellulose and are composed of elementary fibrils (~2 nm)] at the scale of ~2 to 100 nm (*13*, *14*). Constructing even smaller ion transport pathways at the ångström scale requires the ability to manipulate the linear cellulose molecular chains, which are polysaccharides consisting of several hundreds to many thousands of linked anhydrous glucose units (AGUs). These highly hydrophilic polymeric chains are tightly packed through a massive hydrogen bonding network because of the large population of hydroxyl groups (*12*). Although methods for disrupting this hydrogen bonding network and thereby manipulating the cellulose molecular chains have been reported by us and others (*15*), no studies had shown an ordered, crystalline cellulose structure for ion transport applications, particularly at the ångström scale where useful properties and behavior may be observed (*7*, *16*).

Here, we demonstrate a facile, scalable, sustainable, and cost-effective process for synthesizing a cellulose-derived supramolecule (CS) for fast ion transport. In this approach, the intramolecular hydrogen bonding network is broken and the cellulose polymeric chains are reconstructed into a different crystalline structure by coordinating copper ions to the O2 and O3 sites of the cellulose AGUs with the help of hydroxide (OH^−^) and Na^+^ ions. This molecular engineering strategy creates a 3D, crystalline, and hierarchical scaffold that is composed of highly aligned, 1D, and ångström-scale open channels of ~10 Å in diameter (Fig. 1B), through which ions (e.g., Na^+^) can travel rapidly and unidirectionally in aqueous conditions. In this Na^+^-containing cellulose supramolecular (i.e., “Na-CS”) framework, the cellulose molecular chains act as ligands, while the copper ions serve as the metal nodes, critically providing good structural stability. The synthesis is universal to a variety of cellulose materials, including wood, textiles, fiber, paper, ink, and gel. The resulting Na-CS adds to the supramolecule family (*17*), in addition to opening a range of applications. For example, unlike previous cellulose-derived materials, Na-CS displays superior structural stability at high pH (e.g., 14 or higher) (*12*), allowing for the utilization of cellulose in devices with strong alkaline environments, such as alkaline batteries, fuel cells, and electrolyzers (*18*, *19*). Wood-based Na-CS (synthesized from delignified wood to take advantage of the naturally aligned wood vessel channels) shows high ionic conductivities in NaOH solutions of medium to high concentrations [e.g., 0.23 S/cm when soaked with 20 weight % (wt%) NaOH at 25 °C] even with a dense microstructure, enabled by the ångström-scale open channels. This phenomenon is distinct from conventional membranes that generally rely on large pores (submicrometers to a few micrometers) to achieve such high ionic conductivities. The high ionic conductivity of Na-CS makes it highly promising for biological (e.g., ionic cables, sensors, and diagnostics) and electrochemical applications (e.g., selective and ion-conductive membranes), where fast ion transport through a structured medium is desired (*20*–*27*).

## RESULTS AND DISCUSSION

**Fig. 2.**
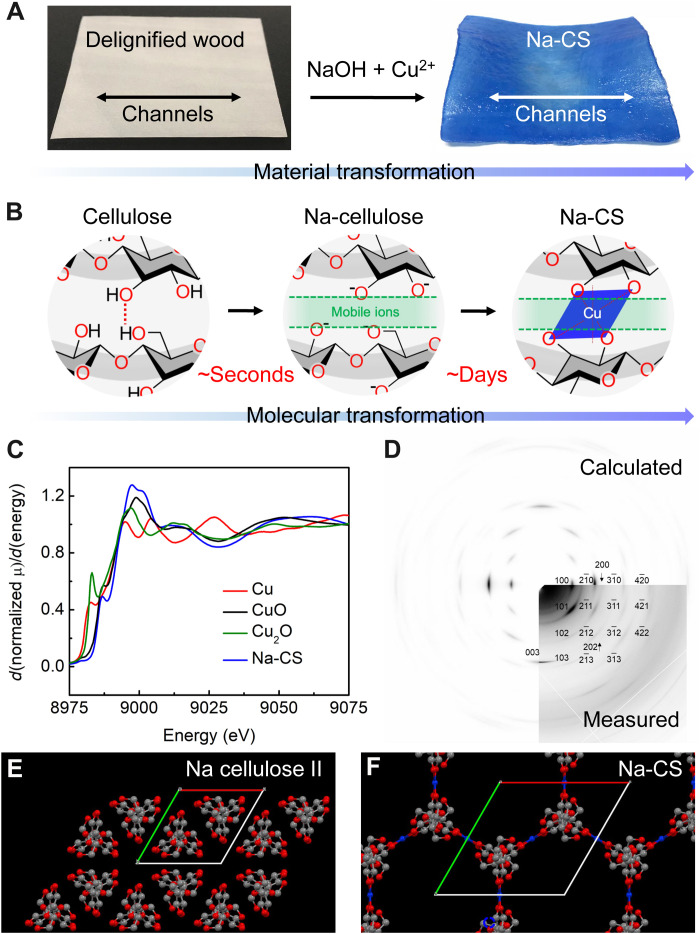
Fabrication and structural characterization of Na-CS. (**A**) Digital images showing the process of transforming delignified wood (white) as a model cellulose material to Na-CS (blue), which demonstrates a gradual color change over the course of ~1-week immersion in Cu^2+^-saturated NaOH solution. (**B**) Schematics showing the transformation at the molecular level, including the fast deprotonation and opening of the cellulose molecular chains upon the formation of Na-cellulose complex, and the slower coordination of the cellulose molecular chains to the copper ions. (**C**) Cu K-edge XANES and EXAFS spectra of Na-CS, Cu foil, CuO, and Cu_2_O. (**D**) Measured and calculated XRD patterns of Na-CS, showing excellent agreement. (**E**) Na cellulose II crystal. (**F**) Na-CS crystal. The unit cells are labeled in (E) and (F). (E and F) Both viewed along the axial direction of the cellulose molecular chains.

In a typical synthesis, we immerse copper wires in a 20 wt% NaOH solution for approximately 1 week until Cu^2+^ reaches saturation (see the Supplementary Materials for experimental details). We then soak the cellulose materials in this Cu^2+^-saturated alkaline solution to complete the Na-CS synthesis, in which they gradually change to a dark blue color (figs. S1 and S2). This process can be applied to a range of cellulose materials, such as paper, textiles, fiber, and wood. To study the ion transport properties of Na-CS, we used delignified wood (in which lignin and hemicellulose have been removed to obtain high-purity cellulose) (*28*) as a starting material to take advantage of its naturally aligned vessel channels. The resulting wood-based Na-CS (Fig. 2A) retains the original aligned, hierarchical, and porous structure of the delignified wood, as observed by scanning electron microscopy (SEM) (fig. S3) (*28*). At the molecular scale, once immersed, the highly alkaline environment should deprotonate the hydroxyl groups of the cellulose molecular chains, thereby breaking the original hydrogen bonding network (Fig. 2B). The Na^+^ ions can quickly insert into the cellulose molecular chains to form the Na-cellulose complex (*29*, *30*). Meanwhile, we hypothesize that the copper ions diffuse more slowly into the spacing between the cellulose molecular chains (as indicated by the slow, multiday color change of cellulose materials) and coordinate with the deprotonated hydroxyl groups of the cellulose molecular chains to form a complex (i.e., Na-CS) (Fig. 2B).

We use X-ray absorption near-edge structure (XANES) and extended X-ray absorption fine structure (EXAFS) to characterize the formed complex. Our results show that the electronic state of the copper ions in Na-CS is mostly similar to that of CuO but differs at ~9000 and ~9060 eV, likely due to the differences of the surrounding chemical environment (e.g., the presence of Na^+^ and structural water in Na-CS) (Fig. 2C) (*31*, *32*). The pre-edge peak of Na-CS (8986.8 eV) suggests that the sample is free of Cu_2_O (8983 eV) or Cu (8982.7 eV), with the first-order derivative showing a better match between Na-CS and CuO, suggesting that the copper ions in Na-CS are close to the 2+ state (fig. S4). Fitting of the XANES and EXAFS spectra revealed a Cu-O distance of 1.93 ± 0.01 Å with a Debye-Waller factor of 0.004 ± 0.001 (fig. S5). This distance can be attributed to either one of the equatorial oxygen (O_eq_) in the Jahn-Teller octahedral complex model, O_eq_ in the square pyramid complex model of copper coordination, or the oxygen in the square planar model of the Cu-O coordination environment in Na-CS (fig. S6A) (*33*–*35*). In addition, we found that the coordination number of copper in Na-CS was 3.2 ± 0.3, which is consistent with the aforementioned models. The copper ion strongly coordinates with four oxygen atoms from cellulose in a planar configuration (resulting in a coordination number of ~4), in agreement with density functional theory (DFT) calculations (fig. S6B), while it remains loosely associated (and thus may not be reflected by the calculated coordination number) with zero, one, or two additional oxygen atoms in the axial direction (*36*), also corresponding to the aforementioned models.

We used X-ray diffraction (XRD) to further characterize the structural transformation during synthesis, in which we observed ~60 peaks in the fiber diffraction pattern of Na-CS (see Supporting Discussion I: Structural Analysis, table S1; fig. S7). This is significantly different from that of the delignified wood starting material, which displays a cellulose Iβ structure (fig. S8). To identify the Na-CS structure, we constructed different crystal models, calculated their theoretical fiber diffraction patterns, and compared with the experimental results to identify the best match, thereby determining the most possible 3D molecular structure. Among all the scenarios we screened, we found one particular structure (fig. S9) based on the *P*3_2_21 space group with a threefold symmetry (fig. S10) that matched well with the experimental diffraction pattern for nearly all the peaks in terms of their position and intensity (Fig. 2D). Note that such a highly ordered crystalline structure in aqueous conditions (Fig. 1B) is markedly different from what we have recently reported with a similar composition of Cu and cellulose, which was achieved through solvent exchange (from water to dimethylformamide) followed by drying to be amorphous in a solid state (*37*). The solvent (i.e., water) and the alkaline condition together play a critical role here in maintaining the highly ordered structure with the massively aligned channels, which can induce unique ion transport properties (*3*–*6*).

In this selected model, the cellulose transforms from cellulose Iβ to Na cellulose I and Na cellulose II driven by NaOH, and gradually to the Na-CS structure after copper chelation (fig. S11) (*15*). In particular, the cellulose molecular chains in the Na cellulose II crystal (Fig. 2E) rotate and spread out to accommodate the inserted copper ions, opening up spaces between the cellulose molecular chains to form the final Na-CS product (Fig. 2F) (see details in Supporting Discussion I). In this Na-CS structure, the AGUs that are coordinated by copper ions with O2 and O3 of the neighboring cellulose chains (*29*, *30*) form 3_1_ helices along the *c* axis (i.e., along the molecular chains). The copper ions form [CuO_4_] motifs in Na-CS with oxygen atoms from the neighboring cellulose molecular chains while weakly coordinating with two additional oxygen atoms from two water molecules perpendicular to the [CuO_4_] plane, agreeing with the Jahn-Teller octahedral complex model (fig. S6). After reorganizing the cellulose molecular chains via copper chelation, Na-CS forms a 3D, hierarchical, and supramolecular structure, composed of massively aligned, 1D, and ångström-scale open channels with a diameter of ~10 Å (Fig. 1B, structure in 3D, and fig. S9, structure in 2D). With this structure, we also calculated the 1D diffraction profile using atomic coordinates based on the model we built with a *P*3_2_21 space group (fig. S10) and with the consideration of preferred orientation of neighboring cellulose chains due to the shape of the cellulose fiber (see details in Supporting Discussion I, table S2; figs. S12 to S14). Despite slight differences likely due to the state of water molecules and Na^+^ ions, the calculated profile matches the measured diffraction patterns reasonably well, which supports the 3D Na-CS structure (Fig. 1B and fig. S9).

**Fig. 3.**
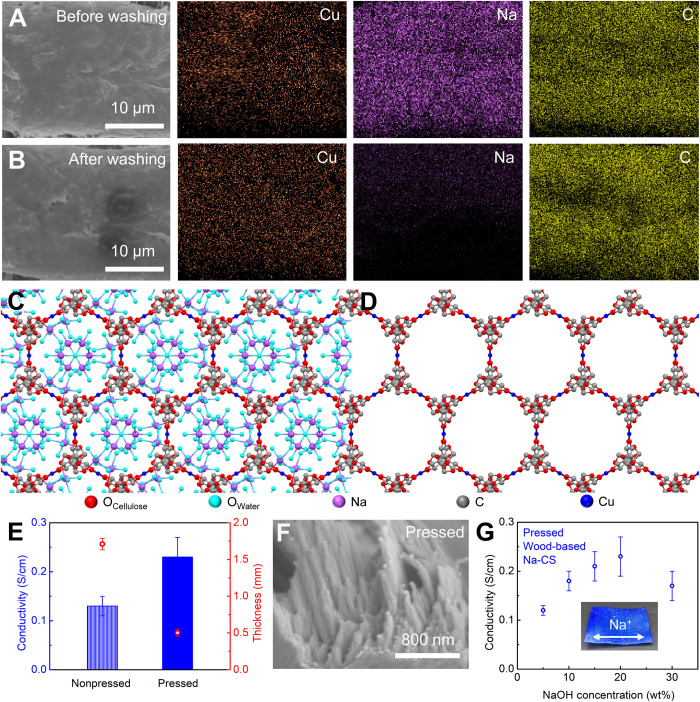
Structure of Na-CS for ion conduction. (**A**) EDS mapping of Na-CS before washing, showing signals of both copper and sodium. (**B**) EDS mapping of Na-CS after washing, in which the sodium signal is barely detectable, while the copper signal remains nearly unchanged. (**C**) Schematic (generated from our model) showing the massively aligned 1D channels, containing Na^+^ ions and the water molecules used to solvate them. (**D**) Schematic of the massively aligned 1D channels where water molecules and alkali ions are removed, while the copper ions remain at the same positions to hold the framework together. (**E**) In 20 wt% NaOH solution, the ionic conductivity of the wood-based Na-CS increases with reduced membrane thickness due to pressing. The nonpressed condition corresponds to the cross-sectional microstructure shown in fig. S17A. The pressed condition corresponds to the cross-sectional microstructure shown in fig. S17B. (**F**) SEM with a perspective view of the pressed wood-based Na-CS, showing that the nanofibers are densely packed. (**G**) Ionic conductivity of the wood-based Na-CS soaked with NaOH solutions at various concentrations. The inset shows the wood-based Na-CS used for the ionic conductivity measurement. The arrow indicates the channel direction.

To determine whether the metal ions in Na-CS (i.e., copper and sodium) are part of the material’s structural motif or free (i.e., removable and/or exchangeable), we use energy-dispersive X-ray spectroscopy (EDS) to map the distribution of copper and sodium in Na-CS before and after washing with deionized water. Before washing, the Na-CS sample shows signs of both copper and sodium with uniform distribution (Fig. 3A). After thoroughly washing the sample, it exhibits nearly unchanged copper but almost negligible sodium signals (Fig. 3B). These results indicate that the copper ions are the metal nodes that hold Na-CS together, while the Na^+^ ions are free and mobile in the structure. Our model suggests that, for each copper ion in Na-CS, up to two water molecules in the axial direction weakly donate two electron lone pairs to the cationic copper center. This means that some of the water molecules present are part of the structural motif of the Na-CS complex, while the remaining water molecules are free and mobile in the porous and hydrophilic scaffold retained from cellulose. Many of these free water molecules can form the solvation shells of the Na^+^ ions because of their strong Lewis acidity. The presence of Na^+^ ions and their solvated water molecules in the Na-CS structure is shown in Fig. 3C according to our crystal structure modeling. The free water molecules (as opposed to those chelated with copper) and their solvated Na^+^ ions reside in the aligned channels of Na-CS, where they can move freely and directionally in the open channels (Fig. 3D).

We hypothesized that the massively aligned, ~10-Å-wide channels of Na-CS would enable unique size and structural effects for ion transport (*3*, *20*, *38*, *39*). For this reason, we used the wood-based Na-CS to study the ion transport behavior, as it also features aligned cellulose fibers along the wood growth direction, making it a highly anisotropic material. Zeta potential measurements show that the delignified wood has a surface charge of approximately −15 mV, while the surface charge of the wood-based Na-CS becomes less negative (approximately −5 mV) upon copper coordination to the hydroxyl groups on the cellulose molecular chains. Using electrochemical impedance spectroscopy (EIS), we measured the ionic conductivities of the wood-based Na-CS soaked with NaOH solutions of various concentrations. We observed that the ionic conductivity of the wood-based Na-CS significantly increased from 0.13 to 0.23 S/cm by reducing the membrane thickness from 1.7 to 0.5 mm through physical compression (Fig. 3E and fig. S15), which removes the large pores/channels (figs. S16 and S17) and creates a dense microstructure with tightly packed nanofibers (Fig. 3F). We hypothesize that the reduced porosity of the wood-based Na-CS shifts the ion conduction pathway from the macropores and mesopores of the wood structure to the ångström-scale ion channels of the Na-CS structure, which likely induces the fast ion transport. In general, the ionic conductivity through a solid medium (e.g., a membrane) can be calculated by σ = σ_0_ (*a*/τ), where σ is the apparent ionic conductivity (measured) with respect to the total geometric area, σ_0_ is the effective ionic conductivity (intrinsic), *a* is the effective area ratio defined by the open space over the total geometric area, and τ is the tortuosity. As the effective area for ion conduction is much smaller than the total geometric area due to the use of structural material as well as its tortuosity (*40*), conventional membranes (e.g., glass fiber) typically need to have a highly porous structure and/or a low tortuosity to achieve reasonably good ionic conductivity. In stark contrast, despite having a dense microstructure, the wood-based Na-CS still demonstrates high ionic conductivities with a much lower porosity compared to the glass fiber (fig. S18 and table S3) because of the presence of the unique ion-conducting channels.

Note that it is critical that there are copper ions stabilizing the Na-CS structure over a wide range of NaOH concentrations (Fig. 3G), without which the cellulose materials cannot form structurally stable membranes, necessary for conductivity measurement or device application at highly alkaline conditions. In addition, we expect that, with a lower and lower concentration of NaOH, we should observe a plateau region where ionic conductivity remains relatively constant with varied salt concentrations, which would show that the ionic conductivity is governed by the surface charge density in the channels (*41*). However, Na-CS requires relatively high pH (high OH^−^ concentrations, e.g., ≥1 mol/L) to maintain its ordered structure, which prevents the exploration on the surface charge–governed ion transport by reducing the salt concentration.

The fast ion transport can be attributed to the unique structure of the ångström-scale open channels. It has been shown that membranes with subnanometer pores can incur unique transport mechanisms of certain solute species (*42*). In particular, the transition state theory suggests that the energy barrier for solute transport through membranes with subnanometer pores largely depends on the pore size and shape, electrostatic repulsion, dielectric effects, weak van der Waals forces, frictional and viscous effects, and any combination of the above factors (*43*). This highlights the importance of being able to engineer the pore and channel structures as well as surface properties at the molecular level toward fit-for-purpose applications. To understand the ion transport mechanism in the Na-CS structure, we use Born-Oppenheimer molecular dynamics simulations (see details in Supporting Discussion II). Our results suggest that a surface-hopping mechanism occurs in the 1D channels dictating the Na^+^ ion diffusion along the axial direction, in which Na^+^ ions can dynamically exchange positions between the solution phase in the middle of the 1D channel (i.e., get coordinated with water and/or OH^−^) and the cellulose/electrolyte interface (i.e., get coordinated with cellulose, water, and/or OH^−^). Figure S19 shows the observed instantaneous coordination number of a typical hopping event. This result demonstrates the directionality of the diffusion pathway (i.e., from one cellulose site to another) and the importance of Na^+^ ion exchange between the cellulose/electrolyte interface and the solution phase in the middle of the channel. Among all the Na^+^ ions studied in the model (i.e., Na^+^ ions with different positions in the channel), we found that the ions that move appreciably faster than the others tend to have higher liquid phase coordination numbers (i.e., coordinated with water or OH^−^) and do not interact strongly with the cellulose molecular chains (fig. S20). In other words, Na^+^ conduction is higher in the middle of the channels (where Na^+^ ions are fully solvated by water or OH^−^) than near the cellulose chains (where Na^+^ ions are partially solvated and thus trapped by the cellulose chains). Therefore, we hypothesize that the fast ion transport enabled by Na-CS is likely induced by (i) an appropriate amount of water molecules and OH^−^ solvating and carrying the Na^+^ ions in the channels and (ii) the improved directionality of charge carrier diffusion by the nanoconfinement effect in the Na-CS framework. That is, these channels are small enough for directional ion transport along the channels instead of allowing them to move around randomly, while they are large enough to host water molecules and OH^−^ needed to solvate Na^+^ ions.

**Fig. 4.**
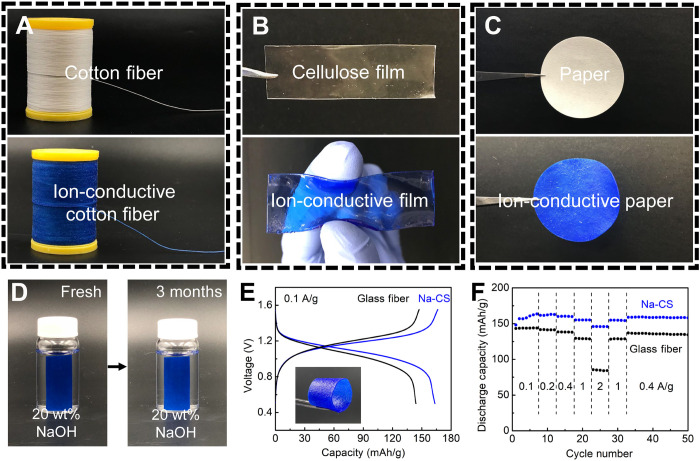
Synthesizing other forms of Na-CS in cellulose materials and their application. (**A**) Cotton fiber (top) and Na-CS fiber (bottom). (**B**) Transparent cellulose film (top) and Na-CS film (bottom). (**C**) Filter paper (top) and Na-CS paper (bottom). (**D**) Digital images of a Na-CS paper before and after soaking in 20 wt% NaOH solution for 3 months. No change of morphology or color was observed, indicating Na-CS’s good stability. (**E**) Comparison of the discharge and charge profiles as well as (**F**) the rate performance of aqueous sodium-ion batteries fabricated using filter paper-based Na-CS and glass fiber as membranes. The aqueous sodium-ion battery featured Ni(OH)_2_ as the cathode and PAQS as the anode. 1 C corresponds to 0.2 A/g_PAQS_.

Our molecular engineering approach can be applied to other salt systems as well. For example, using the same process with 20 wt% KOH instead of 20 wt% NaOH, we synthesized wood-based K-CS, which demonstrated an ionic conductivity of 0.23 S/cm in 20 wt% KOH. This value is higher than the conductivity of wood-based Na-CS in 15 wt% NaOH (0.21 S/cm), where the molarity values of two salts in the solutions are comparable. Furthermore, our approach of synthesizing the Na-CS structure is universal to a variety of cellulose materials. In addition to the delignified wood, we demonstrate numerous forms of Na-CS synthesized from cellulose-based fiber, film, paper, ink, gel, and textile, most of which are derived from wood or other natural materials (Fig. 4, A to C, and fig. S21). Unlike conventional cellulose materials that exhibit poor structural integrity under high pH (e.g., ≥14), the incorporation of copper ions ensures the stability of Na-CS in highly alkaline conditions for several months or even years without noticeable degradation (Fig. 4D and figs. S22 and S23). The large material scope and good stability of Na-CS potentially enables a broad range of applications beyond those related to fast ion transport. For example, we hypothesize that Na-CS can be used in biological, energy, and catalysis fields similar to conventional MOFs and other supramolecular materials by taking advantage of their unique structures and the functionality of the metal nodes (*44*–*48*). Notably, we calculate the materials and manufacturing cost of Na-CS to be as low as ~$1/kg because of the use of cost-effective raw materials and simple processing. The cost of Na-CS is substantially lower compared to conventional MOF materials (*49*, *50*), which is a major benefit of Na-CS for large-scale production and commercialization (*51*, *52*). Overall, our molecular engineering strategy translates to a simple yet scalable and cost-effective process that is universally applicable to cellulose materials.

To leverage these merits and demonstrate its utility, we applied Na-CS as a membrane in an aqueous battery to take advantage of its high ionic conductivity and good alkaline stability. We fabricated the aqueous battery using a Ni(OH)_2_ cathode and poly(anthraquinonyl sulfide) (PAQS) anode in 20 wt% NaOH electrolyte (*53*). In this application, we used a sheet of filter paper as the cellulose material to prepare the Na-CS membrane (fig. S24), which has a high cellulose content and features a fiber-based structure with similar random orientation as the glass fiber separators commonly used in this application. Under different charge/discharge rates of 0.5 to 10 C (where 1 C equals a current density of 200 mA/g_PAQS_), the battery featuring the Na-CS membrane exhibits higher capacities (Fig. 4E) and much better rate capabilities (Fig. 4F). Beyond aqueous batteries, the application of Na-CS can be expanded to many other areas, such as in biosensing, nanofluidic devices, ionotronics, ionic thermoelectrics, and water purification, where fast ion transport is needed (*11*, *20*, *21*, *54*).

In conclusion, we synthesized an ordered, 3D, and anisotropic cellulose supramolecule (i.e., Na-CS) composed of massively aligned, 1D, and ångström-scale open channels. This is achieved by opening the cellulose molecular chains and reordering them with Na^+^ and chelated copper ions to form a previously unidentified aligned channel structure that is ~10 Å wide. These ångström-scale channels in Na-CS enable high ionic conductivities across a wide range of NaOH concentrations, distinct from conventional membranes that typically rely on large pores to conduct ions. In addition, Na-CS is structurally stable in highly alkaline environments owing to the presence of copper ion nodes, which is significantly different from conventional cellulose materials under these conditions. Our molecular engineering synthesis approach is generally applicable to a range of cellulose materials including wood, cotton textile, cotton thread, paper, film, ink, and gel. This study demonstrates a facile yet highly scalable, sustainable, and cost-effective approach for fabricating ion transport pathways at the ångström scale for many applications.

## MATERIALS AND METHODS

### Preparation of delignified wood

NaClO_2_ (Sigma-Aldrich) was dissolved in deionized water to obtain 5 wt% NaClO_2_ solution, whose pH was adjusted to ~4.6 using acetic acid (Sigma-Aldrich). Wood pieces (e.g., basswood, Walnut Hollow Company) were then immersed in the boiling NaClO_2_ solution until the samples turned completely white (i.e., forming delignified wood). The delignified wood was then washed with deionized water three times to remove any residual chemicals.

### Preparation of CS

A Cu^2+^-saturated NaOH (Sigma-Aldrich) solution was prepared by soaking copper wires (McMaster Carr) in 20 wt% NaOH (*15*). This process gradually turns the solution blue. The Na-CS samples were fabricated by immersing the cellulose materials (delignified wood, filter paper, cotton, textile, film, ink, and gel) into the Cu^2+^-saturated 20 wt% NaOH solution. Typically, the cellulose materials gradually turn blue after soaking in this solution within ~14 days. The process is deemed complete after the sample color does not change further or the solution color stops fading. The preparation of K-CS is the same as the preparation of Na-CS, except using 20 wt% KOH (Sigma-Aldrich) instead of 20 wt% NaOH.

### Fiber XRD measurement

XRD measurements were conducted at beamline A1 at the Cornell High Energy Synchrotron Source (CHESS) synchrotron facility of the Wilson Synchrotron Laboratory, Cornell University. The wavelength and sample-to-detector distance was calibrated with a CeO_2_ standard with FIT2D 18.002 version software. Approximately 60 independent peaks up to (0 0 12) can be indexed in the XRD pattern as listed in the Supplementary Materials. In-house fiber XRD measurements were conducted on a Xenocs Xeuss SAXS/WAXS system with a Cu Kα (λ = 1.5418 Å) microfocus source and Dectris Pilatus 300k detector in transmission mode. A special sample holder was used to bring samples close to the SAXS detector.

### XANES and EXAFS measurements

XANES and EXAFS measurements were conducted at the Materials Research Collaborative Access Team (MRCAT), Sector 10-BM beamline (*55*) of the Advanced Photon Source at Argonne National Laboratory. Measurements were conducted in transmission geometry using 20-cm gas ionization chambers filled with gas mixtures suitable for the incident beam (5 to 10% absorption) and the transmitted beam (80% absorption) at the Cu K-edge (8979 eV). Data were collected in continuous scanning mode with a Cu metal foil as reference. Data were processed with the Athena and Artemis programs from the IFEFFIT suite (*56*, *57*). The data from 2 Å^−1^ < *k* < 12 Å^−1^ were Fourier-transformed using a Hanning window with *dk* = 2 Å^−1^ and *k*-weight = 2 and then fit with a single Cu-O path in the range 1 Å < *R* < 2 Å with the Hanning window of *dR* = 0.2 Å. The amplitude reduction factor *S*_0_^2^ was set to 0.91 from the fit to a standard reference Cu foil.

### SEM and EDS measurements

The microstructure and morphology of the prepared samples were observed by SEM (Tescan GAIA FEG SEM). An X-ray detector (AMETEK Octane Plus) was used to collect the EDS data.

### Porosity measurement

The porosity of the glass fiber and pressed wood-based Na-CS was measured using water to fully infiltrate the pores. The volume occupied by water (derived from the weight change of the membrane before and after water infiltration) is then divided by the total volume of membrane and water. Deionized water (purity > 18 megohm cm^−1^) is selected here because of its appropriate wettability with both materials.

### Zeta potential measurement

The zeta potentials of delignified wood and wood-based Na-CS were measured using the Malvern Panalytical zetasizer. To prepare the dispersions for measurements, a piece of delignified wood or wood-based Na-CS was placed into a glass vial with deionized water. The glass vial was vigorously sonicated overnight to ensure a homogeneous dispersion state of the sample for accurate measurement.

### Ionic conductivity measurement of the wood-based CS

The wood-based Na-CS synthesized from delignified wood was used for the conductivity measurements shown in Fig. 3. To ensure the measurement of the ionic conductivity along the channels, the electrical current was applied in the same direction of the wood channels (i.e., along the wood growth direction). Before the measurement in NaOH solutions, the wood-based Na-CS was thoroughly soaked in the testing solution to equilibrate with the testing solution. The wood-based Na-CS was then extracted from the soaking solution for the ionic conductivity test by gently wiping off the free liquid from the surface. To measure the ionic conductivity of the samples, Pt foils were used as the electrodes, which were attached to the two sides of the wood-based Na-CS at various lengths. A two-electrode configuration was used. EIS was then performed over a frequency range of 1 MHz to 100 mHz with a 20 mV perturbation amplitude. To exclude the contact resistance, EIS measurements on the samples with at least three different lengths were performed, where an extrapolation of slope was used to calculate the conductivity based on the following equation: *R*_measured_ = *R*_bulk_ + *R*_contact_ = *L*/σ*S* + *R*_contact_, where *R* is the resistance, *L* is the length, *S* is the surface area, and σ is the conductivity. The conductivity tests were all conducted on a potentiostat (VMP3, Biologic) at 25 °C. Each of the wood-based Na-CS samples was tested in nonpressed and pressed conditions, with the nonpressed condition corresponding to the natural microstructure with large open pores and the pressed condition corresponding to a dense microstructure in which the natural wood pores were collapsed. The pressing was achieved by using densified delignified wood as the starting material, which was subsequently hot-pressed at 60 °C under ~5 MPa. Before the conductivity measurement after soaking, the wood-based Na-CS was further hand-pressed to reduce the swelling effect and further minimize the porosity. SEM images were taken on the aforementioned microstructures after freeze-drying the samples. The ionic conductivity measurement of K-CS is the same as that of Na-CS, except using 20 wt% KOH instead of NaOH solutions.

### Cell fabrication and testing of the aqueous batteries

The electrochemical performance of aqueous batteries was evaluated using a two-electrode coin cell (CR2032) configuration. PAQS anode was synthesized using a previously reported procedure (*58*). PAQS, Ketjen black carbon, and polytetrafluoroethylene were mixed in a mass ratio of 6:3:1 with the aid of ethanol. The resulting mixture was pressed onto a stainless steel current collector (316 type; 100 × 100 mesh) and dried under vacuum at 80°C overnight. The areal mass loading of the PAQS was ~10 mg cm^−2^. The Ni(OH)_2_ cathode was fabricated by pressing the Ni(OH)_2_ powder onto a Ni foam with an areal loading of ~50 mg cm^−2^. The Ni(OH)_2_ cathodes were precharged to 50% of full capacity using activated carbon cloths as the counter electrode before being coupled with the PAQS anodes in full cells. The capacity of the full cells was limited by the PAQS anodes. Glass fiber (Whatman, GF/B) and the Na-CS membrane made of filter paper (Fisherbrand Plain Filter Paper Circles, P8 grade) were used as separators to compare the rate and cycling performances. Both separators were soaked in 20 wt% NaOH electrolyte before cell assembly. The battery tests were all conducted on a potentiostat (VMP3, Biologic) at 25 °C.

### Born-Oppenheimer molecular dynamics modeling

Four unique configurations of a 1 × 1 × 2 supercell (14.9 Å × 14.9 Å × 30.5 Å) of Na-CS were considered at 500-ps intervals from simulations using the OPLS/AA force field. In these configurations, all O2, O3, and O6 sites were deprotonated. An initial thermalization (5 ps) and equilibration period (24 ps) was discarded during which 8 to 25% of the O6 sites were reprotonated, slightly reducing the H_2_O:Na^+^ ratio. This is likely due to the inhomogeneous distribution of cations throughout the cell and reflects local charge compensation effects. An alternative set of trajectories prepared with O6 sites fully protonated each had a higher overall energy than the deprotonated configurations after geometry optimization, indicating that these configurations were less stable. The time scale of the DFT simulations is too short to estimate the equilibrium distribution of protons between the solution and O6 sites and may also be highly dependent on the local environment as alluded to previously. The complete deprotonation of the O2, O3, and O6 sites were taken from the literature (*59*). The cells contained 6 Cu^2+^, 28 Na^+^, and 4 OH^−^ (36 negative charges from cellulose and 4 OH^−^ balancing 6 Cu^2+^ and 28 Na^+^). One hundred twenty-six water molecules were present, which corresponds to a H_2_O to Na^+^ ratio of 4.5 (NaOH·4.5H_2_O) inside Na-CS. All spin-polarized calculations were carried out using CP2K v6.1 (*60*) at 393.15 K using a 0.5-fs time step in the NVT ensemble. To improve the quality of water dynamics (*61*), the revPBE-D3BJ functional was used in combination with the DZVP-MOLOPT-SR-GTH basis set and GTH-PBE pseudopotentials with a cutoff of 1200 Rydberg (Ry) (rel_cutoff was 50 Ry) (*62*–*67*). Mean square displacements and coordination environment were measured in each of the four trajectories over the final ~25 ps from a ~49-ps simulation. Thermostatting was provided by a single Bussi velocity rescaling thermostat with a time constant of 50 fs (*68*). Analysis was supported by the Atomic Simulation Environment and MDAnalysis (*69*, *70*). Visuals were prepared with Jmol (*71*).

A second set of simulations with a H_2_O:Na^+^ ratio of 9:1 (NaOH·9H_2_O) was prepared from OPLS-AA simulations at 2.5-ns intervals, with only O2/O3 sites (the Cu^2+^ ligands) deprotonated. The 14.9 Å × 14.9 Å × 30.5 Å (angles: 90°, 90°, and 120°) supercell was used here as well. A geometry relaxation to the default convergence criteria in CP2K was performed to relieve strain in the structure related to differences between force field– and DFT-preferred properties such as bond lengths and bond angles. After relaxation, a 3-ps initial heating phase was performed in the NVT ensemble to a target temperature of 273.15 K (Bussi global thermostat; 11 fs time constant). Approximately 20 ps of simulation was also performed at 333.15 K, but the temperature was further increased after no significant displacement (>5 Å^2^) was measured. The cutoff has been lowered to 600 Ry to expedite trajectory generation with minimal cost to accuracy relative to the previous setup. The data collection phase was performed with a target temperature of 393.15 K, with other settings being identical to the previous phase. Proton exchange was not observed in this set of simulations; thus, equilibration time was limited to the first 10 ps. CP2K version 8.2 was used for this set of trajectories. The revPBE-D3BJ functional and DZVP-MOLOPT-SR-GTH + GTH-PBE basis and pseudopotential set are used here as well. Spin polarization is also considered here.

## Supplementary Materials

### This PDF file includes:

Figs. S1 to S24 Tables S1 to S3 References
