# Supplementary material for: A cellulose-derived supramolecule for fast ion transport
Source: Sci Adv. 2022 Dec 9;8(49):eadd2031. doi: 10.1126/sciadv.add2031 (PMC9733924; doi:10.1126/sciadv.add2031)
Supplement: Supplementary file 1 — Figs. S1 to S24 Tables S1 to S3 References [file sciadv.add2031_sm.pdf]

Science Advances
AAAS

Supplementary Materials for

A cellulose-derived supramolecule for fast ion transport

Qi Dong *et al.*

Corresponding author: Liangbing Hu, binghu@umd.edu; Tian Li, tianli@purdue.edu

Sci. Adv. **8**, eadd2031 (2022)
DOI: 10.1126/sciadv.add2031

The PDF file includes:

Figs. S1 to S24
Tables S1 to S3
Legends for data S1 to S3
References

Other Supplementary Material for this manuscript includes the following:

Data S1 to S3

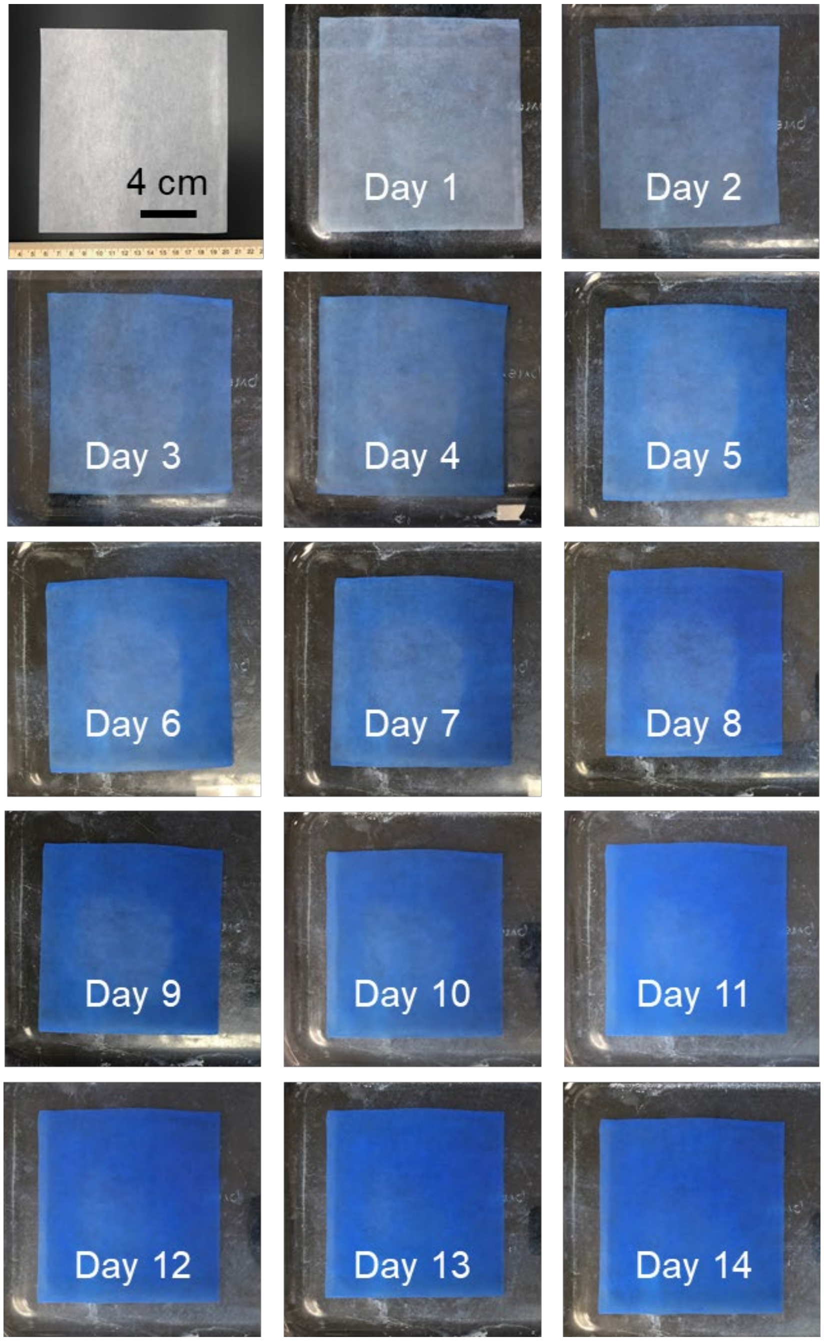

Fig. S1 The color evolution of cellulose paper over 14 days of soaking in the Cu^{2+}-saturated NaOH solution. Paper is used here as a model cellulose material to clearly show the color change.

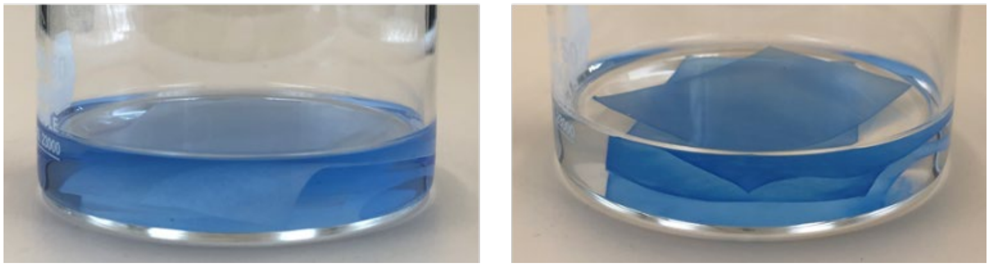

Fig. S2 Digital images showing the transformation from paper to Na-CS. The solution color fades to clear while the color of the paper turns blue.

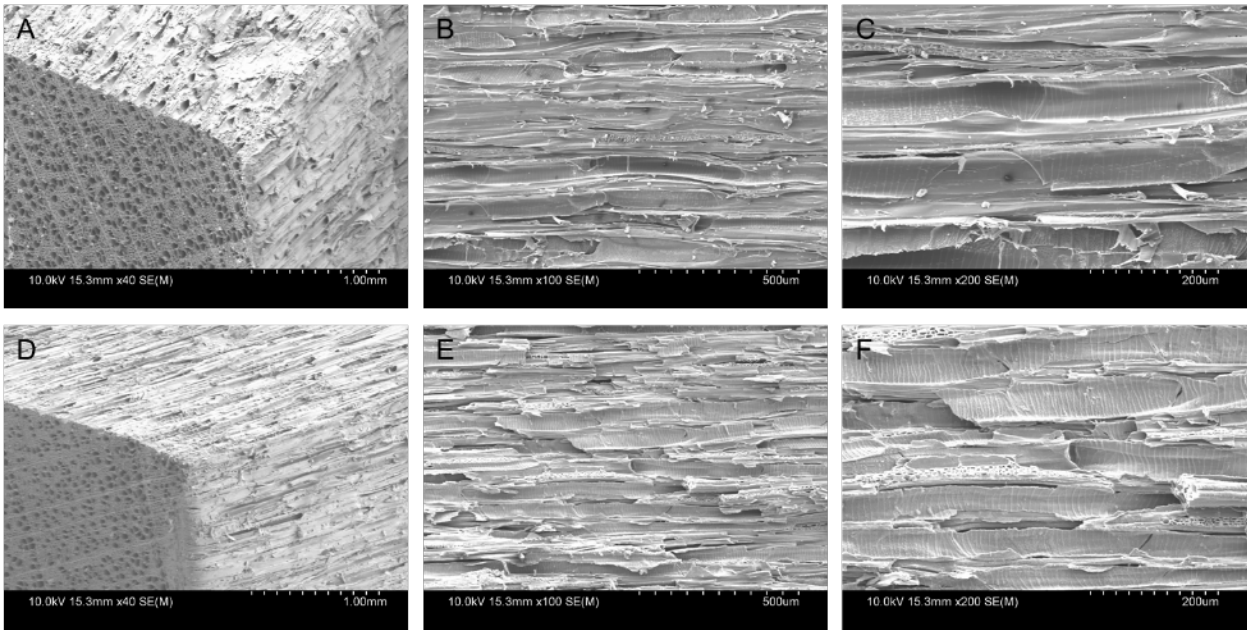

Fig. S3 SEM images of the multiscale structure of Na-CS. The multiscale structure of the (A-C) delignified wood starting material and (D-F) the corresponding wood-based Na-CS obtained after soaking the delignified wood in the Cu^{2+}-saturated 20 wt% NaOH solution for a week. The hierarchical structure, including the aligned channels formed by the wood vessels, is preserved after the synthesis.

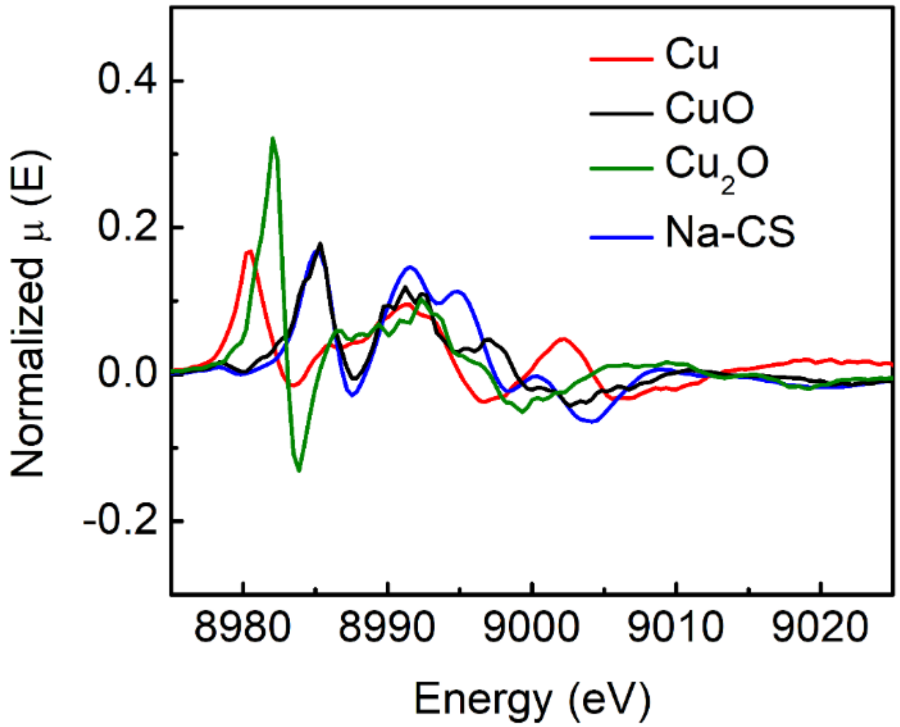

Fig. S4 The first derivative of the Cu K-edge XANES spectra of Na-CS, Cu foil, CuO, and Cu_2O.

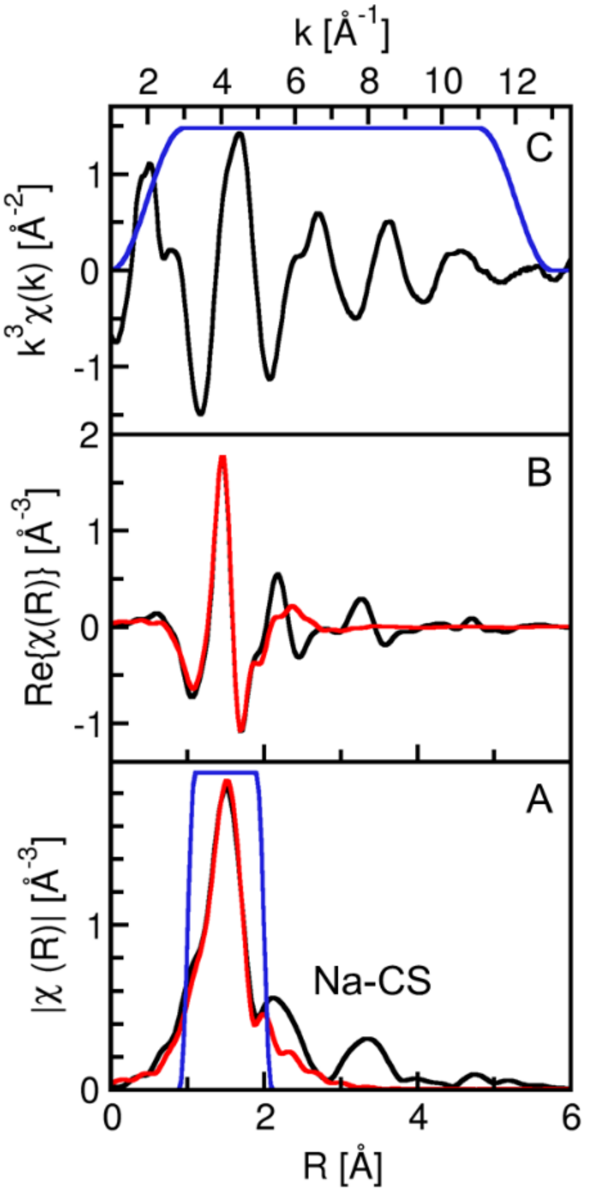

Fig. S5 Fitting of the XANES and EXAFS spectra of Na-CS at the Cu K-edge (A) The magnitude of real space χ(R). (B) The real part of χ(R). (C) The χ(k). Fitting parameters were R_{Cu-O} = 1.93 ± 0.01 Å, σ^2 = 0.004 ± 0.001, and N_{Cu-O} = 3.2 ± 0.3 with S_0^2 = 0.91. Original spectrum: black lines; first shell fit: red lines; and window functions: blue lines.

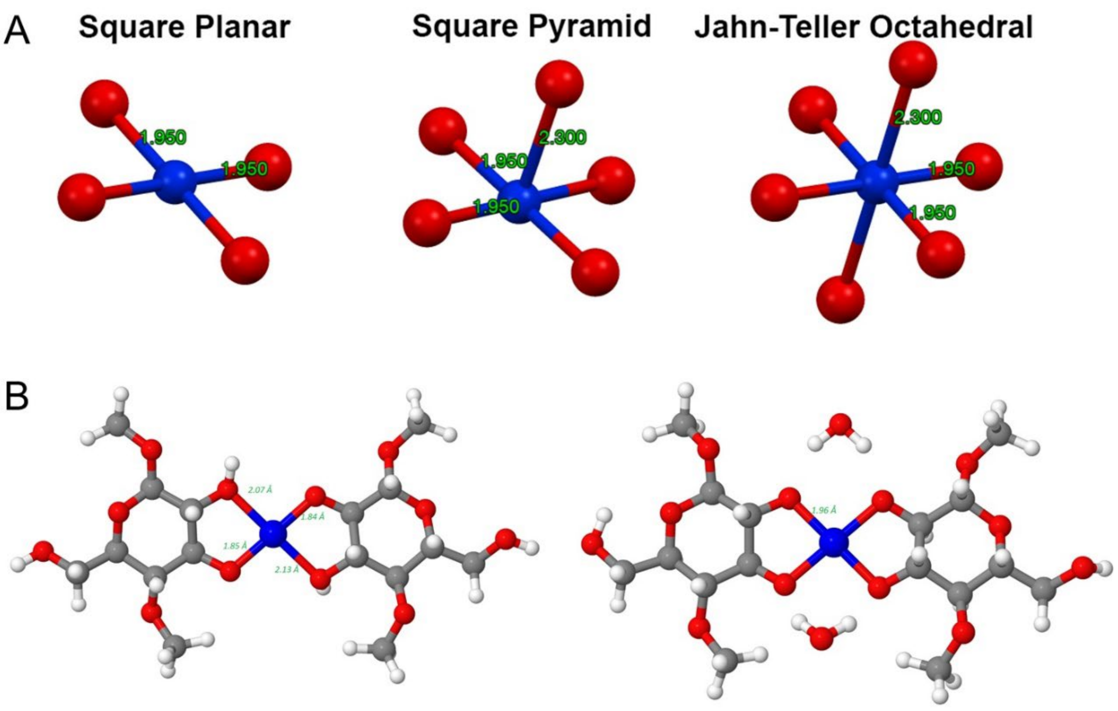

Fig. S6 Coordination environment of the copper ions in Na-CS. (A) Models of possible Cu-O coordination in Na-CS. The copper atom is shown in blue and the oxygen atoms are shown in red. The Cu-O bond distance in the equatorial direction is shown as 1.95 Å in all models and the Cu-O bond distance in the axial direction of the Square Pyramid and Jahn-Teller Octahederal models is shown as 2.3 Å. All bond angles are 90˚. (B) The copper-ion-coordinated cellulose fragments from ωB97XD/6311+G(2d,p) DFT calculations using Gaussian 16 software.

Supporting Discussion I: Structural analysis

We built models to analyze the structure of the Na-CS by positioning atoms within the unit cell in reasonable ways until we found the best match of the calculated XRD patterns with the experimental data. The cellulose chain conformation is restricted by the AGU rigidity. As a result, only limited combinations of AGU-copper complex positions are permitted within the constraint of the unit cell. We built models for all combinations of the permitted handedness, direction of chains, and position of copper ion, calculated the theoretical fiber diffraction pattern of each possible configuration using a computer program we wrote, cross checked with XPolar and Mercury software, and selected the model that fit the XRD and XANES data the best. The modeled Na-CS structure can be achieved by simply rotating the cellulose chains in the Na Cellulose II structure reported by Atkins *et al.* (*29*) by 60˚ around the center of the chain in the z direction, then spreading out the rotated molecular chains with enough space, and finally inserting a copper ion.

The model with the copper ion coordinating with the O2 and O3 atoms of neighboring chains was built with a 3-fold symmetry operation reported in the literature (*29, 72*) with slight numerical modification to the bond angles between the neighboring AGU rings along the chain into an accepted range. To semi-quantitatively analyze the quality of the model, a calculation of the 1D XRD pattern based on the $P3_221$ space group in TOPAS with consideration of preferred orientation of the cellulose fiber is given in Fig. S12. The used CIF files are included in the Supporting Information. The dihedral angles at the glycosidic linkage are Φ = -79.45˚ (C5-C1-O1-C4') and ψ = 149.81˚ (C1-O1-C4'-C3'), these two torsion angles are not directly comparable to the native cellulose conformation with a twofold axis. The C1-O4'-C4' bond angle is 116.38˚, the O5-C1-O4' bond angle is 108.43˚, the C2-C1-O4' bond angle is 105.03˚, and the bond length between C1 and O4' is 1.421 Å. All bond angles and bond lengths are within normal bond angle and length

ranges and compared in Table S2. O6 is set to the *gg* conformation in the current model with χ~60° (O5-C5-C6-O6) (*29*). Note that the model based on $P3_221$ symmetry has cellulose chains arranged in anti-parallel packing. A parallel packing model based on P62 symmetry is also possible yet less likely (Fig. S11). The XRD data could not distinguish between the $P3_221$ and P62 symmetries. However, the materials before transforming to Na-CS in this work typically displays an anti-parallel packed Na Cellulose II crystal structure, therefore it can be assumed the anti-parallel packing $P3_221$ model is more probable, as transformation from anti-parallel packing to parallel packing has not been observed in cellulose crystals.

The next part of the modeling study was to find the position of the copper ion. Copper ion coordination with the O2 and O3 of atoms of the cellulose AGU structure has also been reported for the dissolution of cellulose in amine-copper-ion complex aqueous solution (*73*). It has been found that the copper ions bonding to O2 and O3 is indeed the most reasonable scenario (*74-76*).

To analyze the size of the elementary fibril of the Na-CS, a wood-based Na-CS sample was soaked in DMSO to exchange and remove NaOH and water, followed by drying in vacuum under room temperature. The Na-CS structure was preserved after the treatment. Fiber XRD was conducted on the dried sample as shown in Fig. S13. An azimuthal accumulation around the (100) peak is shown in Fig. S14. The full width at half maximal (FWHM) is measured via a Gaussian fitting. The diameter of the elementary fibril is calculated based on the FWHM using the Scherrer equation. The resulted fibril diameter (100 Å) is larger than the reported diameter of the cellulose elementary fiber (20–30 Å), the reason is likely due to the swelling of the nanofibres during copper chelation, plus the effect that two or more Cellulose I elementary fibrils merging to form anti-parallel chain packing during the treatment by the NaOH solution.

Table S1. List of the indexed peaks from the fiber XRD results of Na-CS. * indicates the peaks that cannot be separated from neighboring peaks.

#	h	k	l	measured d (Å)	calculated d (Å)	**δ %**	**δ d (Å)**
1	1	0	0	12.93262	12.9038	**-0.22**	**0.03**
2	1	0	1	9.875491	9.85059	**-0.25**	**0.02**
3	2	-1	0	7.474316	7.45	**-0.33**	**0.02**
4	2	-1	1	6.699594	6.69393	**-0.08**	**0.01**
5	1	0	2	6.571137	6.56456	**-0.10**	**0.01**
6	2	0	0	6.497493	6.45189	**-0.71**	**0.05**
7	2	0	1	5.998218	5.94198	**-0.95**	**0.06**
8	2	-1	2	5.332753	5.32874	**-0.08**	**0.00**
9	0	0	3	5.090644	5.08333	**-0.14**	**0.01**
10	2	0	2	4.935933	4.92529	**-0.22**	**0.01**
11	3	-1	0	4.90667	4.87717	**-0.60**	**0.03**
12	1	0	3	4.740251	4.72957	**-0.23**	**0.01**
13	3	-1	1	4.665856	4.64538	**-0.44**	**0.02**
14	3	0	0	4.330217	4.30126	**-0.67**	**0.03**
15	2	-1	3	4.195751	4.199	**0.08**	**0.00**
16	3	0	1	4.132314	4.13975	**0.18**	**-0.01**
17	3	-1	2	4.1243	4.10859	**-0.38**	**0.02**
18	2	0	3	4.006705	3.99291	**-0.35**	**0.01**
19	3	0	2	3.776443	3.74631	**-0.80**	**0.03**
20	4	-2	0	3.753079	3.725	**-0.75**	**0.03**
21	1	0	4	3.661639	3.65625	**-0.15**	**0.01**
22	4	-2	1	3.633371	3.61861	**-0.41**	**0.01**
23	4	-1	0	3.588497	3.57886	**-0.27**	**0.01**
24	3	-1	-3	3.535303	3.51931	**-0.45**	**0.02**
25	2	-1	4	3.397384	3.39391	**-0.10**	**0.00**
26	4	-2	2	3.37072	3.34696	**-0.71**	**0.02**
27	2	0	4	3.295988	3.28228	**-0.42**	**0.01**

28	1	0	5	2.967391	2.96821	**0.03**	**0.00**
29	2	-1	5	2.829811	2.82262	**-0.25**	**0.01**
30	2	0	5	2.76594	2.75742	**-0.31**	**0.01**
31	5	-2	2	2.765897	2.75965	**-0.23**	**0.01**
32	4	-2	4	2.626805	2.66437	**1.41**	**-0.04**
33	3	-1	5	2.592415	2.58597	**-0.25**	**0.01**
34	0	0	6	2.543737	2.54167	**-0.08**	**0.00**
35	1	0	6	2.496577	2.49375	**-0.11**	**0.00**
36	2	-1	6	2.410729	2.40553	**-0.22**	**0.01**
37	4	-2	5	2.328104	2.35986	**1.35**	**-0.03**
38	5	-2	4	2.347724	2.33821	**-0.41**	**0.01**
39	3	-1	6	2.257921	2.25396	**-0.18**	**0.00**
40	4	0	5	2.216656	2.21627	**-0.02**	**0.00**
41	1	0	7	2.149141	2.14817	**-0.05**	**0.00**
42	2	-1	7	2.089541	2.091	**0.07**	**0.00**
43	2	0	7	2.060654	2.06408	**0.17**	**0.00**
44	3	-1	7	1.989775	1.98914	**-0.03**	**0.00**
45	3	0	7	1.942977	1.9435	**0.03**	**0.00**
46	1	0	8	1.886507	1.88578	**-0.04**	**0.00**
47	2	-1	8	1.846675	1.84675	**0.00**	**0.00**
48	3	-1	8	1.775794	1.77545	**-0.02**	**0.00**
49	0	0	9	1.696034	1.69444	**-0.09**	**0.00**
50	1	0	9	1.679891	1.68002	**0.01**	**0.00**
51*	2	-1	9	1.640745	1.65225	**0.70**	**-0.01**
52*	2	0	9	1.640745	1.63887	**-0.11**	**0.00**
53	3	-1	9	1.601543	1.6006	**-0.06**	**0.00**
54	4	-2	9	1.540778	1.5423	**0.10**	**0.00**
55	4	-1	9	1.533104	1.53147	**-0.11**	**0.00**
56	1	0	10	1.514316	1.51446	**0.01**	**0.00**
57	3	-1	10	1.455204	1.45551	**0.02**	**0.00**

58	3	0	10	1.440214	1.43733	**-0.20**	**0.00**
59	4	-1	10	1.401779	1.40294	**0.08**	**0.00**
60	1	0	11	1.377272	1.37843	**0.08**	**0.00**
61	2	-1	11	1.360795	1.36297	**0.16**	**0.00**
62	3	-1	11	1.334799	1.33353	**-0.10**	**0.00**
63	0	0	12	1.276523	1.27083	**-0.45**	**0.01**
64*	1	0	12	1.249067	1.26471	**1.24**	**-0.02**
65*	2	-1	12	1.249067	1.25274	**0.29**	**0.00**
66*	3	-1	12	1.249067	1.22977	**-1.57**	**0.02**

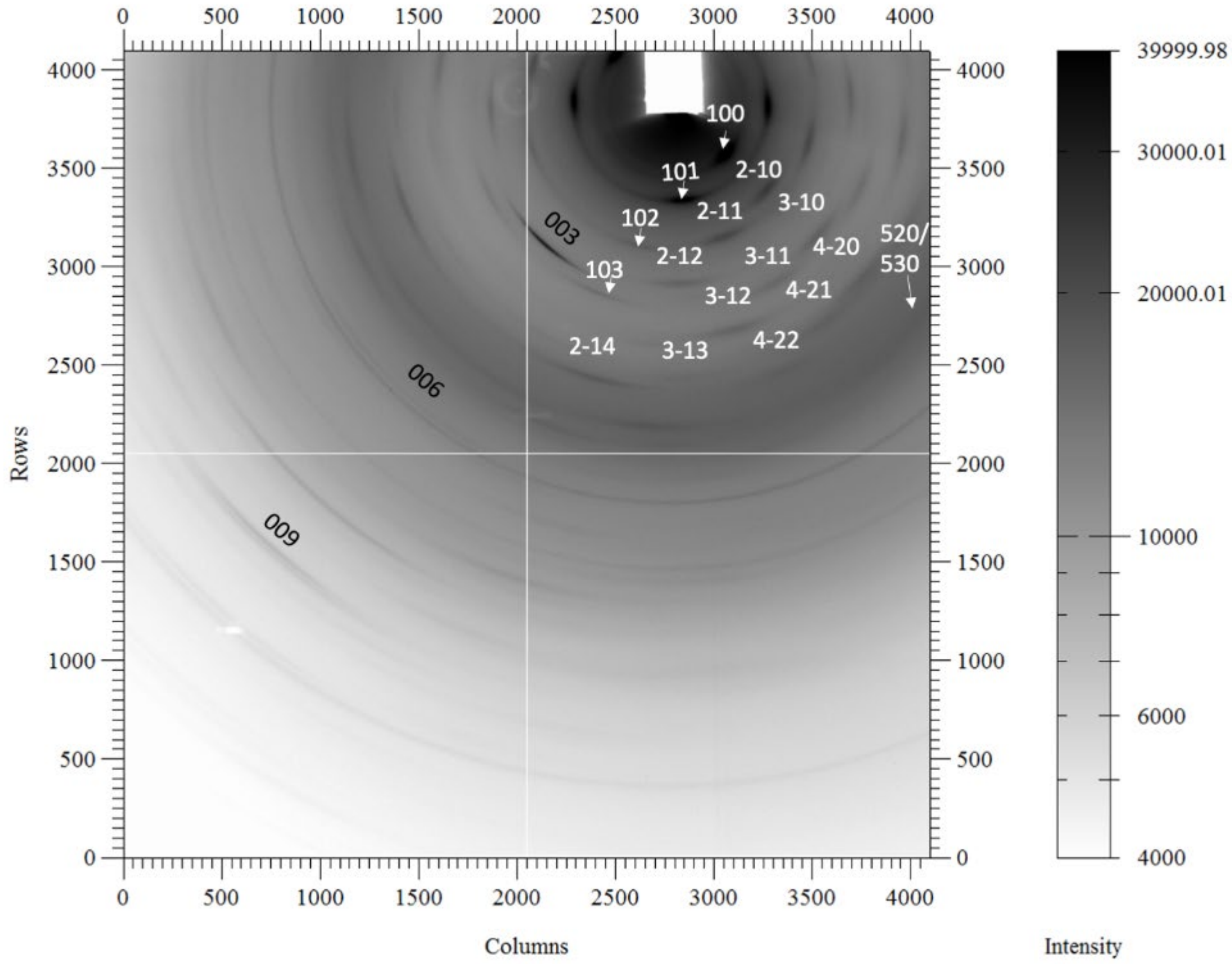

Fig. S7 The full fiber-XRD pattern of the Na-CS, with major peaks indexed.

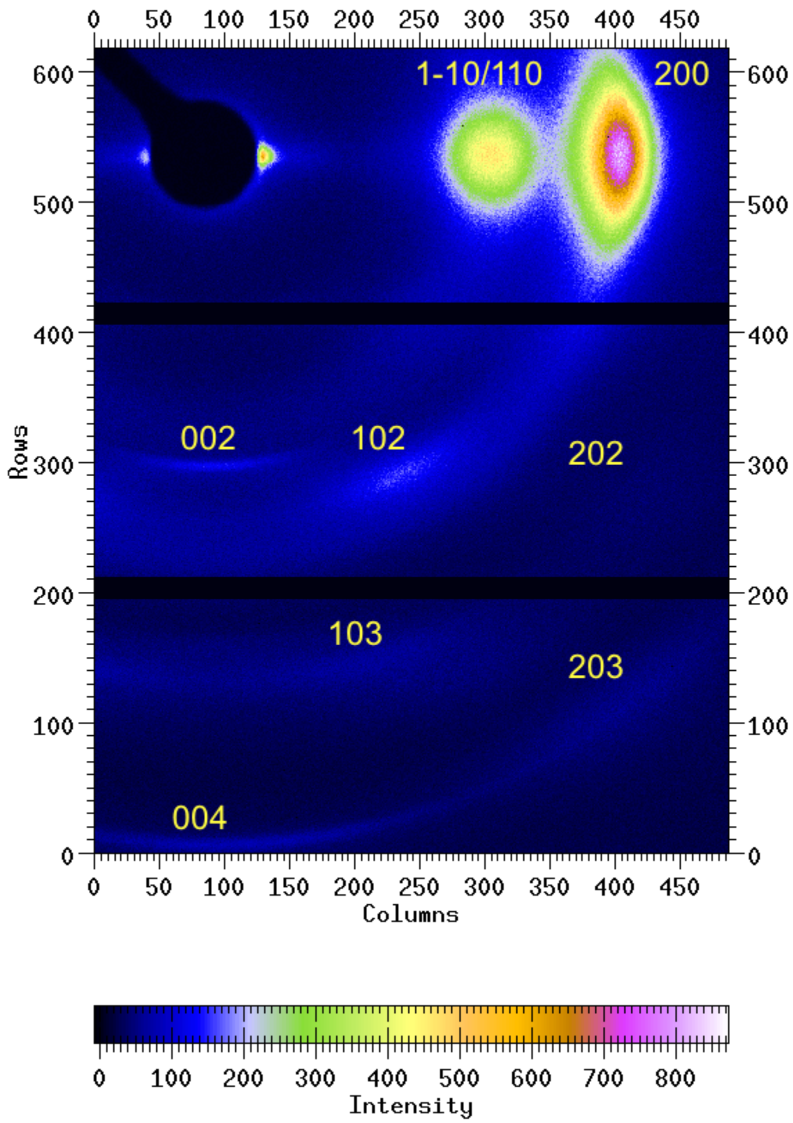

Fig. S8 Fiber XRD pattern of the delignified wood. The XRD pattern is identified as Cellulose Iβ (*29*). The major peaks are indexed on the 2D fiber pattern.

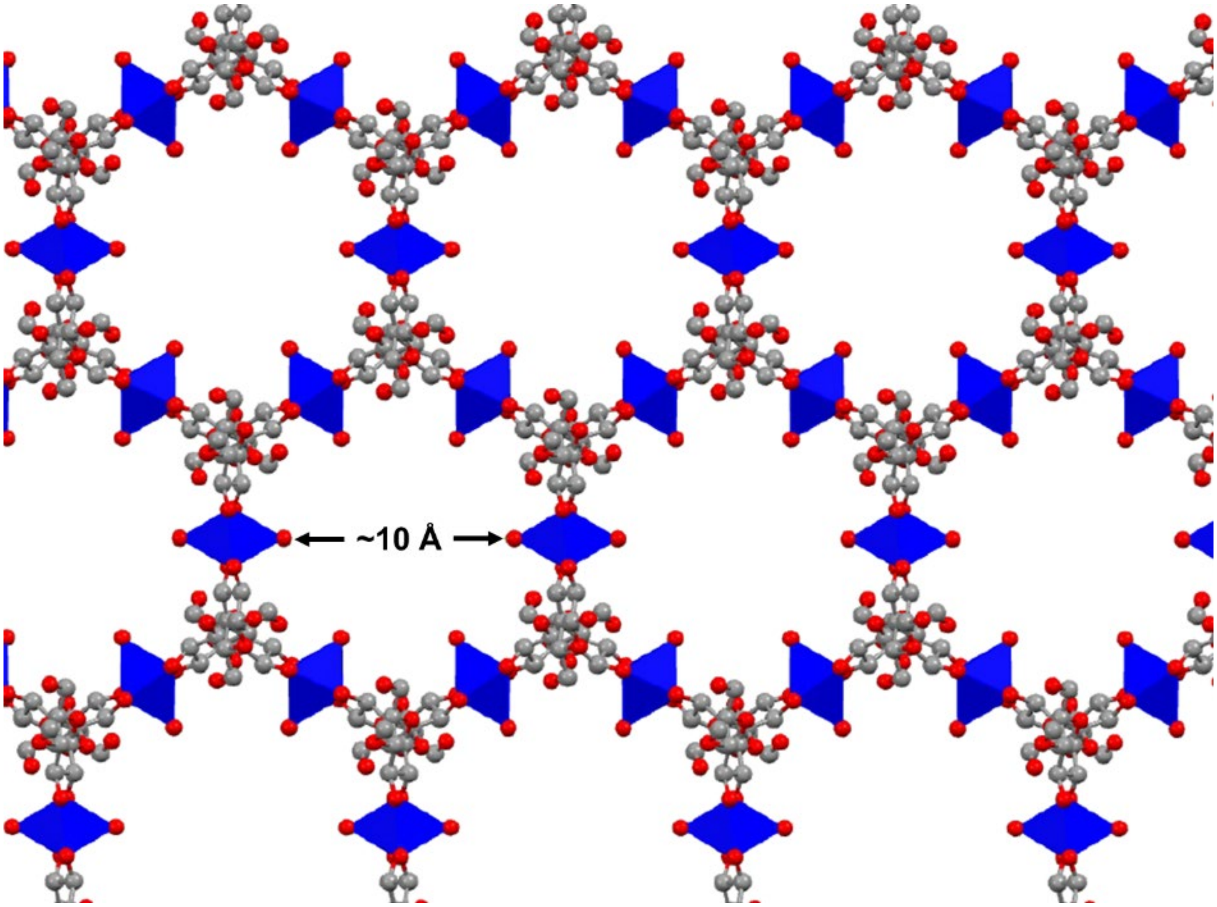

Fig. S9 The molecular structure of Na-CS as viewed along the c direction, *i.e.*, along the cellulose chains and the axial direction of the ion channels. Copper ions coordinate and cross-link all the cellulose molecular chains to form a supramolecular Na-CS structure.

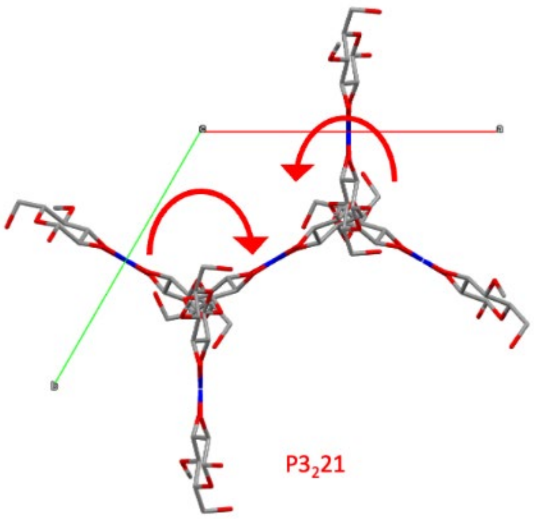

Fig. S10 The difference between the antiparallel packing $P3_221$ symmetry crystal and parallel packing P62 symmetry crystal of Na-CS, viewed from the *c* direction (along the cellulose molecular chains). Arrows indicate the C6-O6 in the gg conformation and used as an indicator of the cellulose molecular chain direction. Color scheme: red refers to oxygen; grey refers to carbon; and blue refers to copper.

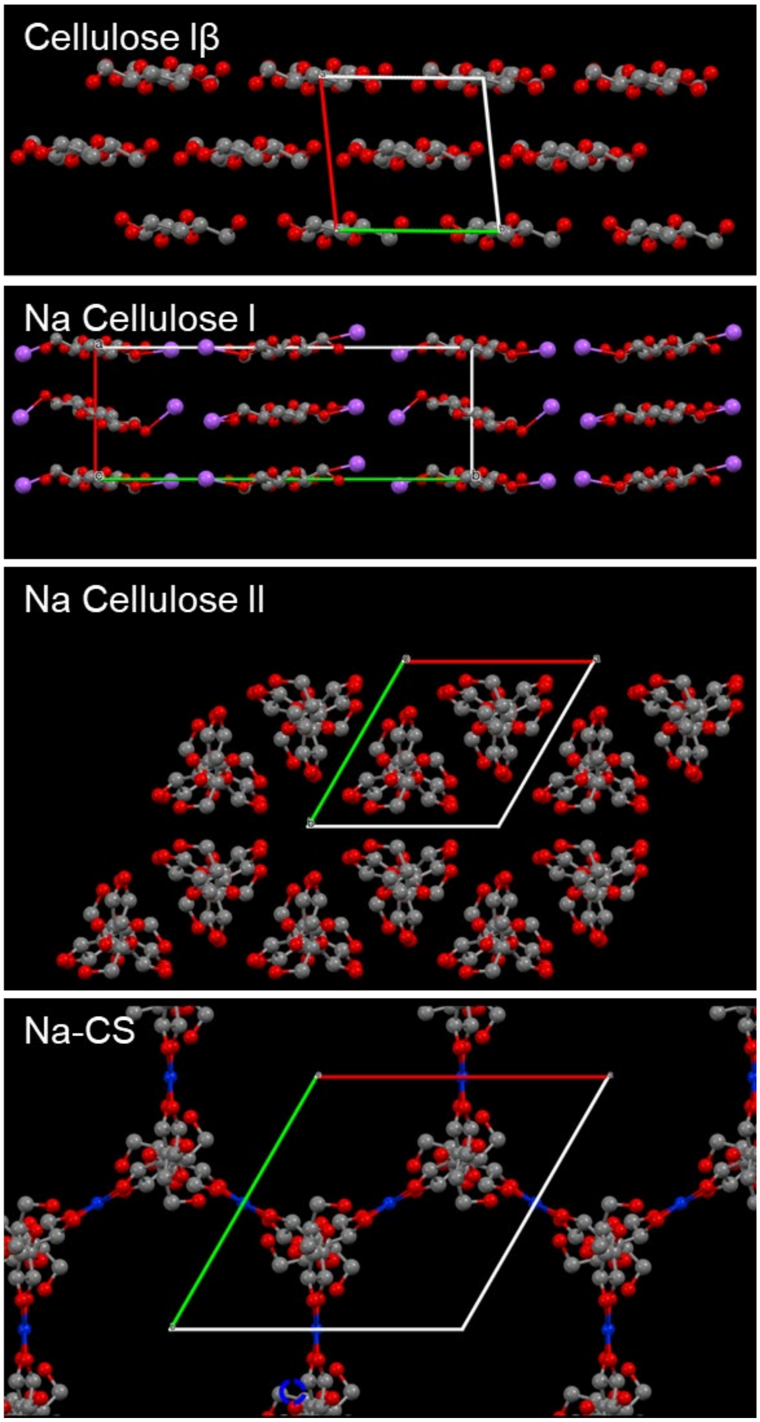

Fig. S11 The molecular structure evolution. The molecular structure evolution from Cellulose Iβ to Na Cellulose I, then to Na Cellulose II, which are driven by NaOH, and finally to Na-CS upon the slow copper insertion (*29, 72*).

Table S2. List of bond angles and bond distances between rigid glycosidic rings from several cellulose allomorphs and Na-CS. The results indicate that the structure of Na-CS is consistent with the reported structures. Structural information in CIF format is available electronically for download.

**Allomorph**	**C1-O4'-C4' angle (˚)**	**O5-C1-O4' angle (˚)**	**C2-C1-O4' angle (˚)**	**C1-O4' distance (Å)**	**C4'-O4' distance (Å)**
Cellulose Iα<br>CCDC 792796 (*77*)	116.17<br>116.06	106.79<br>106.30	105.78<br>107.47	1.397<br>1.409	1.438<br>1.432
Cellulose Iβ<br>CCDC 810598 (*78*)	115.05<br>116.17	106.39<br>105.89	106.67<br>105.39	1.414<br>1.426	1.439<br>1.436
Cellulose II (*79*)	115.62<br>115.09	106.69<br>107.13	108.52<br>106.46	1.393<br>1.394	1.428<br>1.436
Na Cellulose I (*80*)	116.57<br>116.63<br>116.56<br>116.61	106.94<br>106.99<br>106.97<br>106.88	108.30<br>108.39<br>108.25<br>108.33	1.383	1.426<br>1.426<br>1.425<br>1.425
**Modified Na Cellulose II*&**	**116.26**	**106.5**	**104.36**	**1.421+**	**1.421++**
**Na-CS&**	**116.38**	**108.43**	**105.03**	**1.421+**	**1.421++**

+Bond length and other AGU information are taken from the literature (*72*).

++Bond length was set to 1.421 Å as constraint.

*Atom positions are taken from the literature (*29*) with correction of minor printing error and minor adjustment of the AGU ring spatial arrangement.

[&]Chain conformation based on $P3_221$ or P62 symmetry has the same bond angle and bond length, with only the chain direction changed.

The Na-CS was generated by a computer program written by the authors under the following conditions: 1) the AGU ring is rigid; 2) the C4-O4 bond is rigid with a bond length of 1.421 Å; 3) the 5 atoms of O2O3-Cu-O2'O3' are roughly within a plane with Cu-O distance between 1.93 Å to 2 Å due to the fluidic nature of the coordination bond and EXAFS data; 4) the C1-O4' bond length is set to the same as the C4-O4 bond length; 5) $P3_221$ symmetry for arrangement of the AGUs. After the angles between bonds connecting the AGU rings were close to a reported range (115.9°-116.63° for C1-O4'-C4' and 105.78°-108.52° for O5-C1-O4' or C2-C1-O4', 1.383 Å - 1.439 Å for C1-O4' and C4'-O4' distance as listed in Table S2), the authors deemed the structure satisfactory. It should be noted that as the Na-CS is strongly affected by both copper and sodium ions, a larger scale calculation or simulation including all the factors is still needed to fully understand the structure.

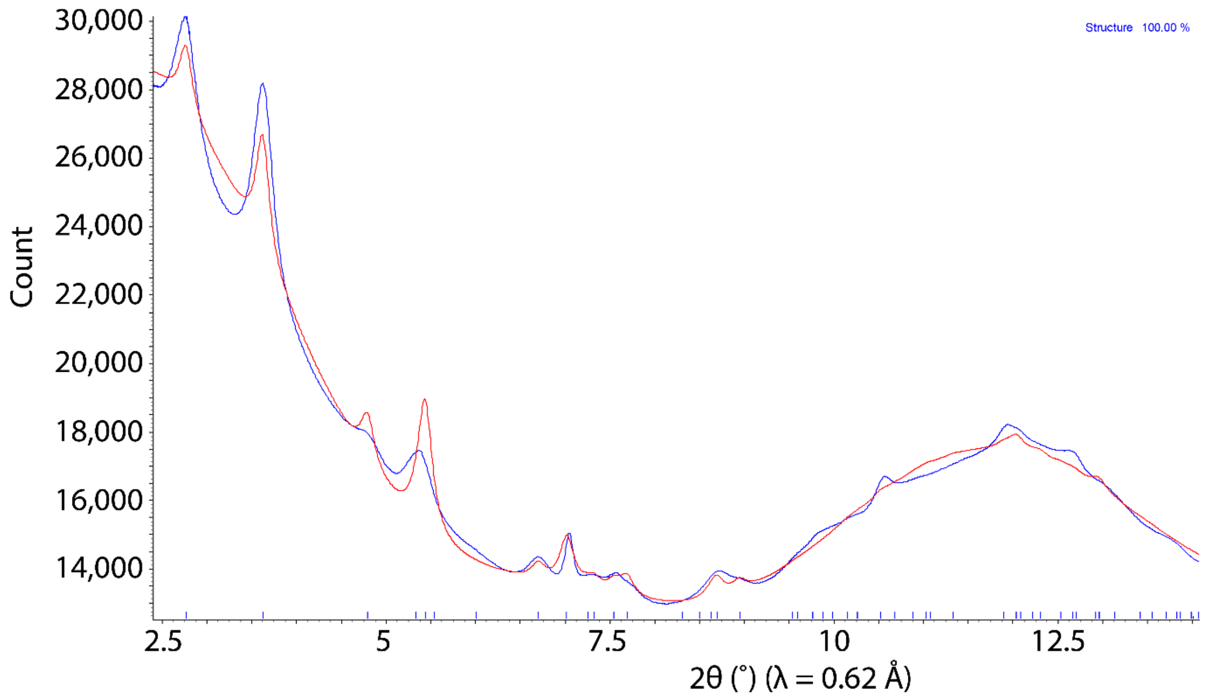

Fig. S12 1D XRD pattern calculation for the Na-CS structure with preferred orientation considered. The experimentally measured XRD data (Figure 2D, Figure S7) is shown in blue, and the calculated diffraction 1D pattern produced by TOPAS (with preferred orientation (001) considered from the $P3_221$ model with water included) is shown in red. The experimental and calculated data show the same trend, indicating the model captures the essence of the structure. The difference between the experimental data and calculation may derive from the position of water and Na^+ as well as the cellulose chain arrangement.

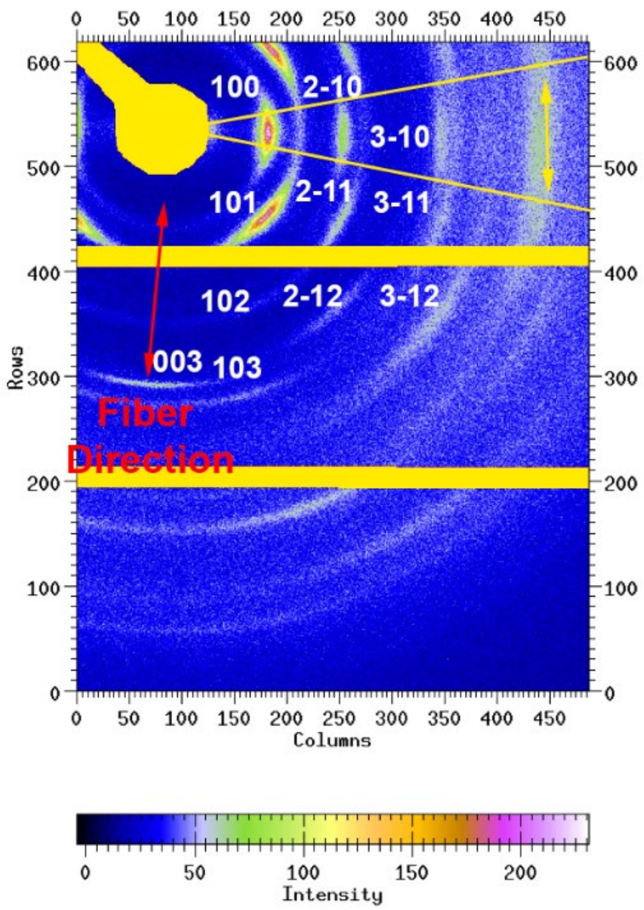

Fig. S13 2D fiber XRD pattern of Na-CS with an azimuthal integration around the (100) peak. The 2D fiber XRD was conducted on a Xenocs Zeuss SAXS system with a copper Kα micro-focusing source. The sample was sealed in an X-ray capillary and scanned in vacuum. Only 1 quadrant was scanned.

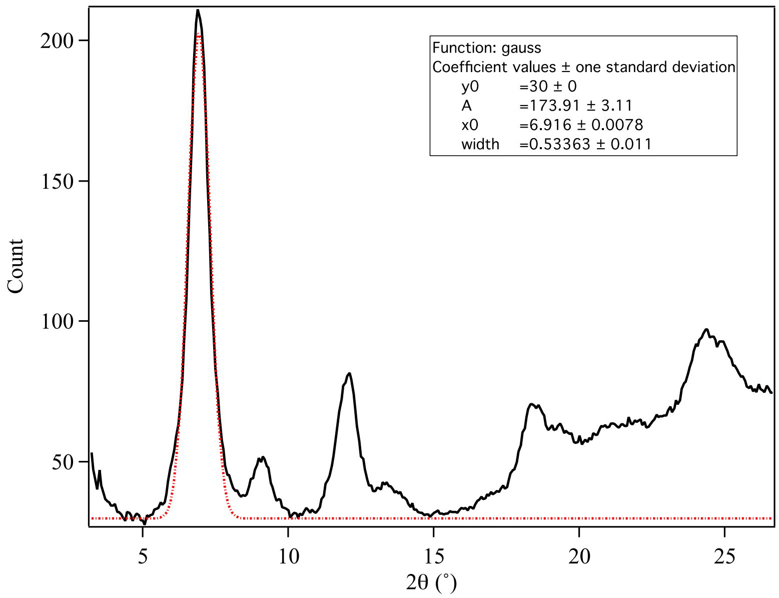

Fig. S14 1D fiber XRD pattern from Na-CS with an azimuthal integration around the (100) peak and a Gaussian peak fitting on the (100) peak as shown in Fig. S13. The fiber diameter can be estimated based on the Scherrer equation as 100 Å, with the shape factor assumed to be 1 and full width at half maximum assumed to be 0.0155 rad from the fitting. The fibril diameter (100 Å) is larger than the reported diameter of the cellulose elementary fiber (20–30 Å), the reason is likely due to the swelling of the nanofibre during copper intercalation, plus the effect of two or more Cellulose I elementary fibrils merging to form anti-parallel chain packing during the NaOH solution treatment.

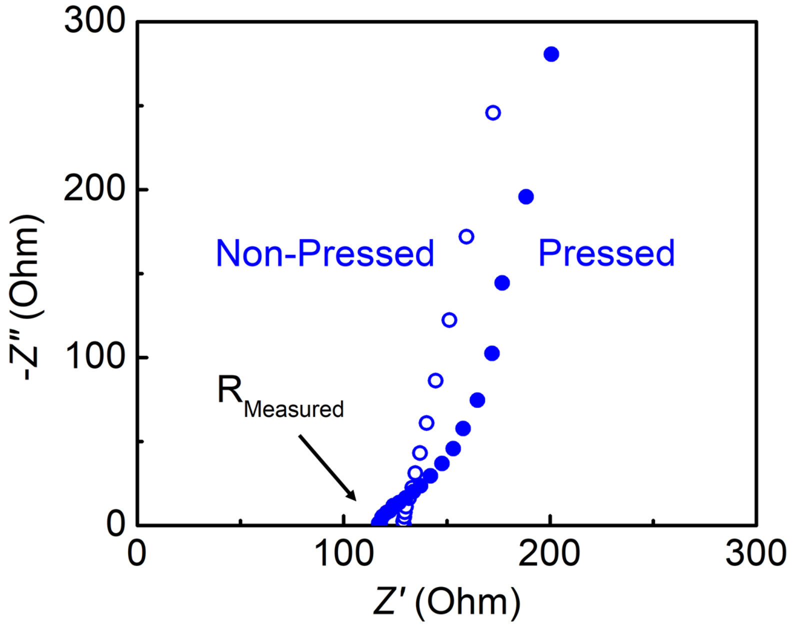

Fig. S15 Representative Nyquist plots of the non-pressed and pressed wood-based Na-CS in 20 wt% NaOH at 25 °C. Due to the complex microstructure of the wood-based Na-CS, the conductivity was extrapolated based on the following equation: $R_{measured} = R_{bulk} + R_{contact} = L/\sigma S + R_{contact}$, where R is the resistance, L is the length, S is the surface area, and σ is the conductivity (see Methods for details).

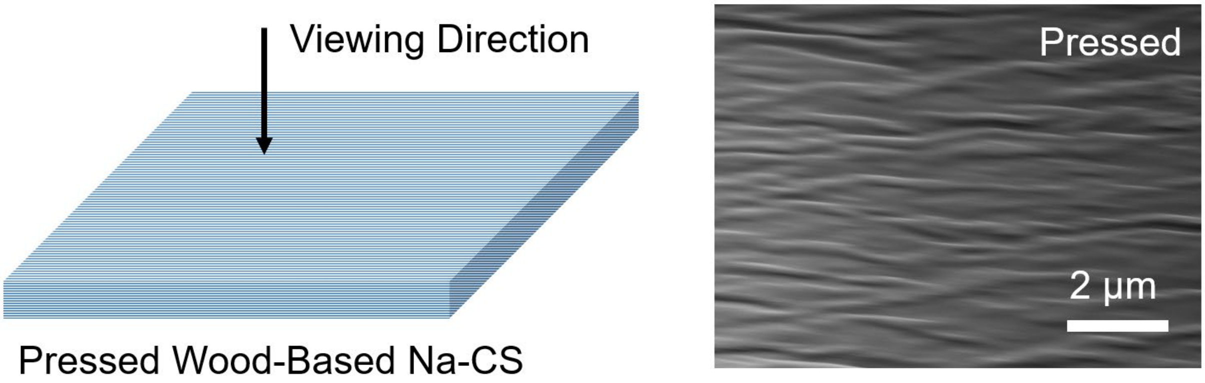

Fig. S16 Schematic and SEM images showing the dense microstructure of the wood-based Na-CS after pressed. The viewing direction is perpendicular to the nanofibres and the channels.

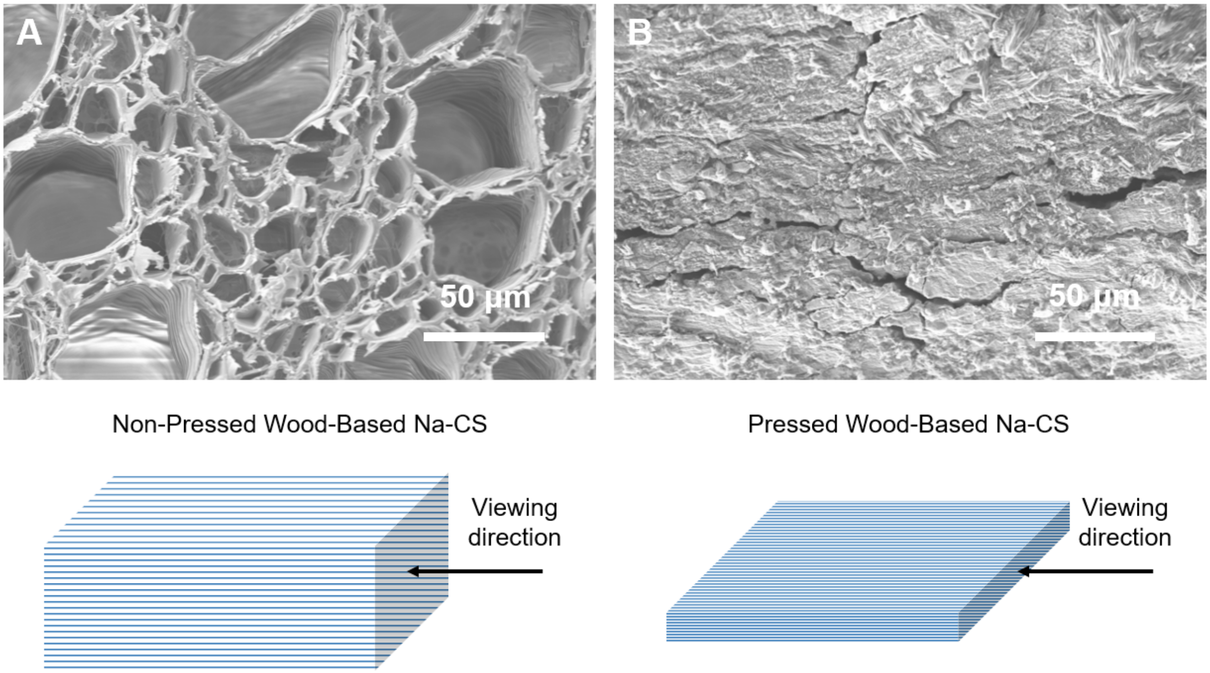

Fig. S17 SEM and schematic images of Na-CS before and after pressing. SEM showing the porous structure of (A) the non-pressed wood-based Na-CS and (B) the dense microstructure of the pressed wood-based Na-CS. The SEM image is viewed along the axial direction of the ion conduction pathways, showing the cross section of the wood-based Na-CS.

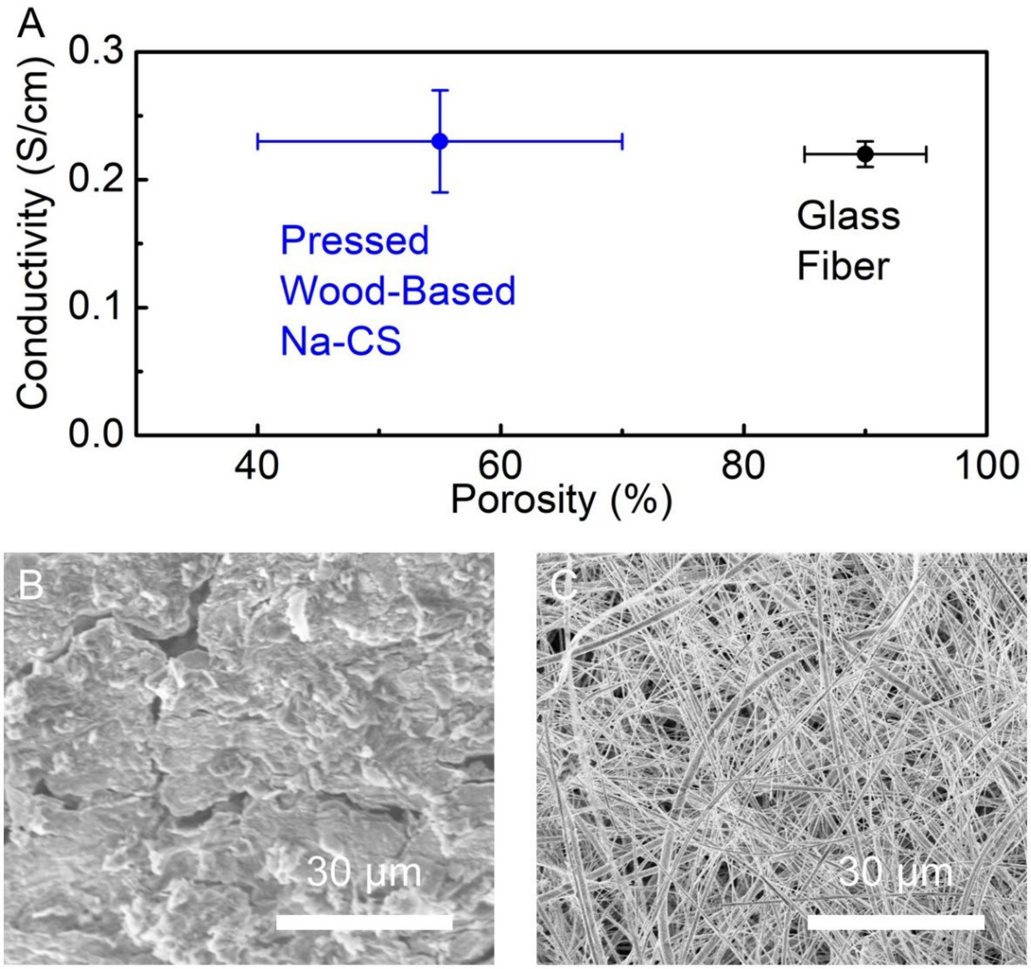

Fig. S18 Comparison between pressed wood-based Na-CS and glass fiber membrane. (A) The pressed wood-based Na-CS exhibits slightly higher ionic conductivity in 20 wt% NaOH solution but much lower porosity compared to the glass fiber. (B) SEM image of the pressed wood-based Na-CS. (C) SEM image of the glass fiber.

Table S3. Comparison of porosity between pressed wood-based Na-CS and glass fiber membrane.

Material	Porosity (%)
Glass fiber membrane	90 ± 5
Pressed wood-based Na-CS	55 ± 15

Supporting Discussion II: Modeling Na^+ transport in Na-CS

Born-Oppenheimer molecular dynamics simulations were performed to explore the diffusion mechanism of $NaOH•4.5H_2O$ and $NaOH•9H_2O$ aqueous solutions inside the Na-CS at elevated temperature (120 ºC). The starting composition of Na-CS considered was fully deprotonated on the O2, O3, and O6 sites for $NaOH•4.5H_2O$ and fully protonated for $NaOH•9H_2O$ (see Methods for details). Because of the computational expense to generate trajectories of ~30-50 ps for these systems, several initial geometries (what we call replicates) are extracted from a series of force field simulations.

The diffusion of Na^+ ions in the Na-CS is highly directional in the direction parallel to the channel. However, we observe that 1D mean square displacements (MSDs) in this parallel direction averaged over the 28 Na^+ ions per trajectory are quite small (< 1 Å) (*72*) over the linear regime. From the slopes of the MSD *vs.* time curves in the time range of 5 ps to 15 ps, the diffusion coefficient of Na^+ ion in Na-CS is estimated to be $2 \pm 0.4\ 10^{-10}\ m^2/s$, which translates to a maximum (ideal) conductivity due to Na^+ on the order of ~45 mS/cm, despite the use of revPBE to improve the dynamic properties of water. For the sake of brevity and because the $NaOH•9H_2O$ trajectories show no appreciable difference from the $NaOH•4.5H_2O$ trajectories, we do not show average MSDs or per particle MSDs for the $NaOH•9H_2O$ trajectories. It may also be the case that OPLS/AA is too aggregating even with scaled charges and the timescale of the DFT simulation even at high temperature is not enough to see dissociation of the ion pairs. Related to the $NaOH•4.5H_2O$ simulations, one unique aspect DFT simulation provides over the force-field-based dynamics simulations, however, is the explicit treatment of the structural diffusion mechanism of OH^- in solution through the $H_3O_2^-$ intermediate described by Tuckerman *et al.* (*81, 82*). Several of these exchanges were observed to occur in the solvation shell of the Na^+ ions immobilized on the

cellulose wall, forming the octahedral complex $RCONa(H_2O)_4OH$. In instances where the coordinated OH^- was converted to H_2O, the resulting $Na^+(H_2O)_5$ species is seen to quickly dissociate from the cellulose. This would suggest that the structural diffusion mechanism of the solvated OH^- plays a role in the exchange of cations between the nano-confined solution phase and the cellulose/electrolyte interface. It is worth noting also that the Cu^{2+} ions remained in place over the length of the simulation further confirming stability of the proposed Na-CS model.

Fig. S19 shows the observed instantaneous coordination number of a typical hopping event. This plot demonstrates the directionality of the diffusion pathway (*i.e.*, from one cellulose site to another) and the importance of Na^+ ion exchange between the cellulose/electrolyte interface and the solution phase in the middle of the channel. By examining the per particle MSD (all 112 ions from all 4 trajectories, Fig. S20), we observed significant heterogeneity of Na^+ displacements on the simulation timescale. The same is true for the case with the $NaOH•9H_2O$ as well, most cations are immobilized (again, relative to DFT timescales) on the anionic sites along the cellulose wall. Fig. S20 examines the correlation between the Na^+ coordination shell composition (x-axis) and the Na^+'s proximity to the cellulose framework, namely the per particle MSD computed to 10 ps (y-axis). The probability that the ion is <3.2 Å away from any of the cellulose oxygen atoms over the length of the simulation is demonstrated using a blue-to-red color gradient. From this figure it is seen that the fastest moving ions tend to have higher liquid phase coordination numbers and do not spend as much time near one of the anionic sites on the cellulose (red dots, upper far right of figure). However, as is demonstrated in Fig. S19, nano-confinement of Na^+ ions in the cellulose channels tends to lead to a more directional diffusion pathway parallel to the channel when large hopping events do occur.

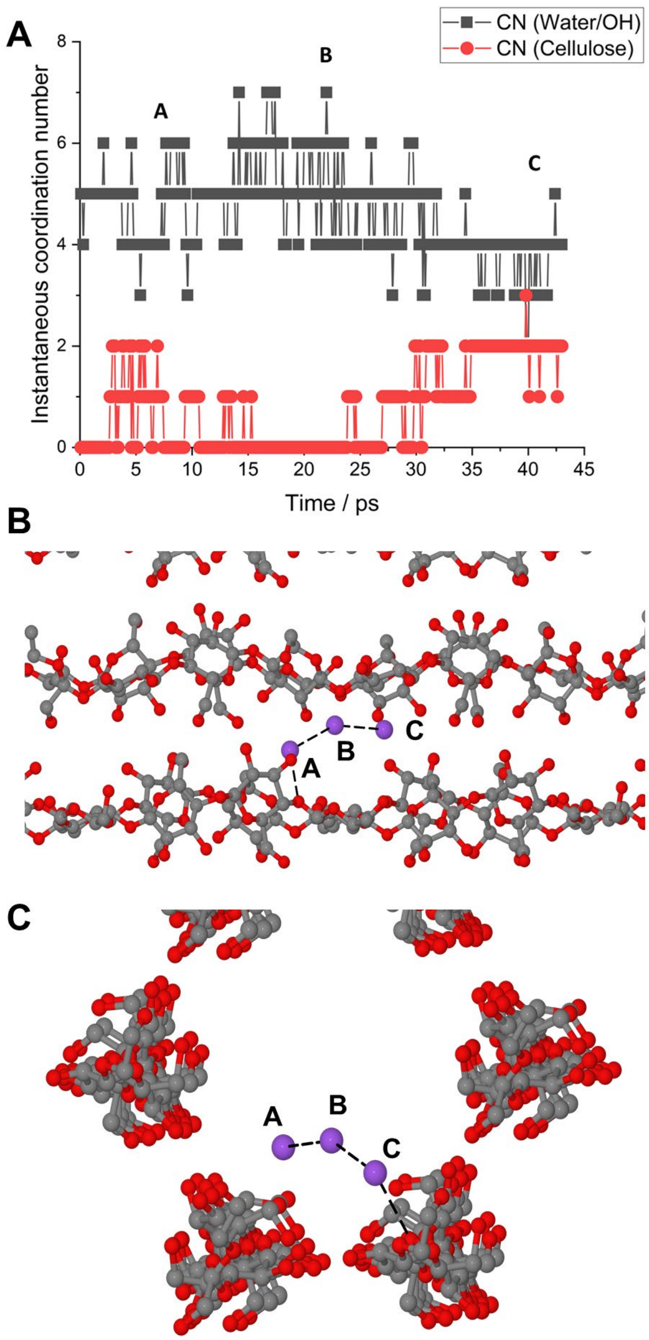

Fig. S19 Born-Oppenheimer molecular dynamics modeling of the Na^+ transport in Na-CS with a NaOH•9H_2O configuration. (A) Instantaneous coordination numbers (Na-O(cellulose) <3.2 Å and Na-O(water/OH^-) <3.2 Å for the most prominent observed diffusion event (largest individual MSD of all ions ~40 Å) (*72*). (B, C) Diffusion pathway inside the channel with labels identifying the coordination environment observed (viewing from different angles). Sodium is shown in purple, carbon is shown in gray, and oxygen is shown in in red. Hydrogens, copper, other sodium ions, and the solvent are omitted for clarity.

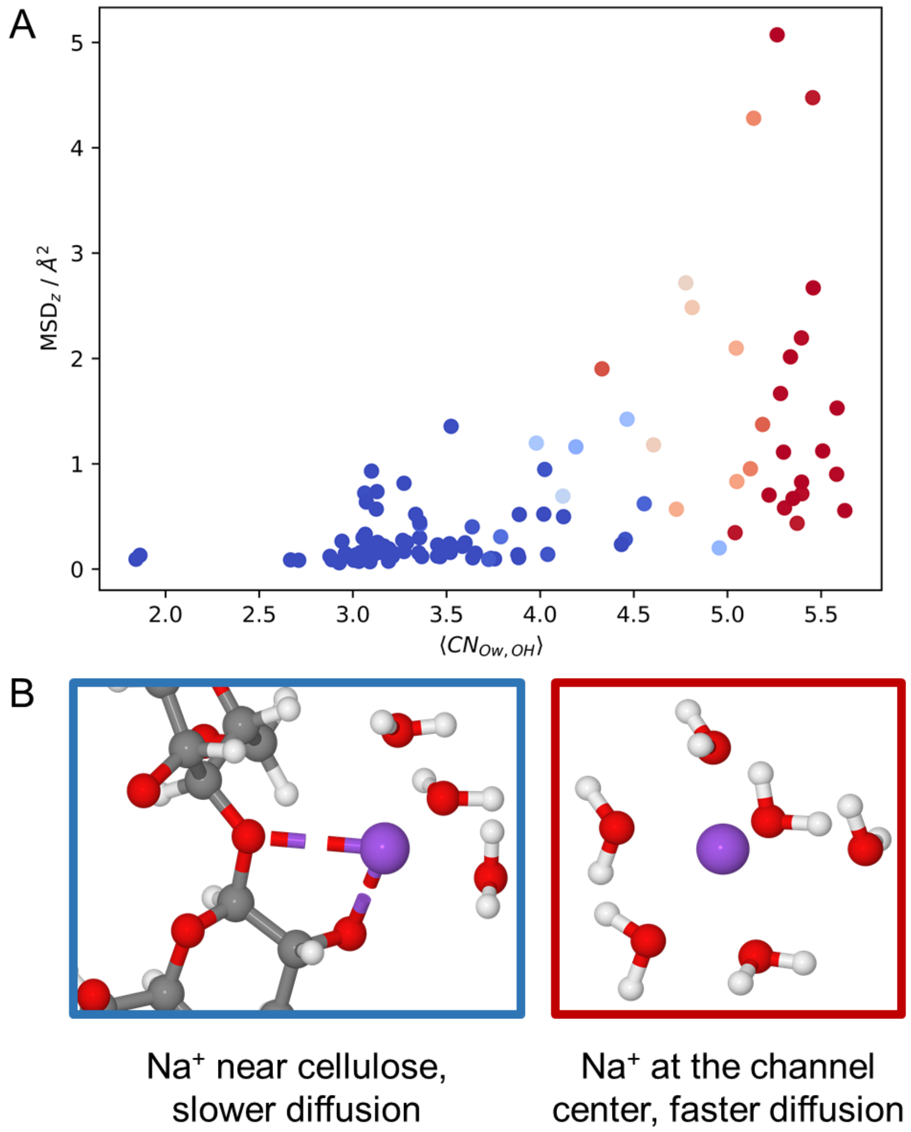

Fig. S20 Born-Oppenheimer molecular dynamics modeling of the Na^+ transport in Na-CS, relating diffusion to the coordination environment of the Na^+ ions. (A) The per particle MSD computed to 10 ps (y-axis) against the average coordination number of Na^+ to water and/or OH^- (x-axis) and probability that the ion is <3.2 Å away from any of the cellulose oxygen atoms over the length of the simulation (color). Blue indicates a high probability to sit <3.2 Å from cellulose oxygen, while red indicates a high probability to reside >3.2 Å from cellulose oxygen atoms. (B) The Na^+ ions near and interacting with cellulose oxygen diffuse slower (left), while those at the channel center, with higher liquid phase coordination numbers and that do not interact strongly with the cellulose molecular chain, diffuse faster (right).

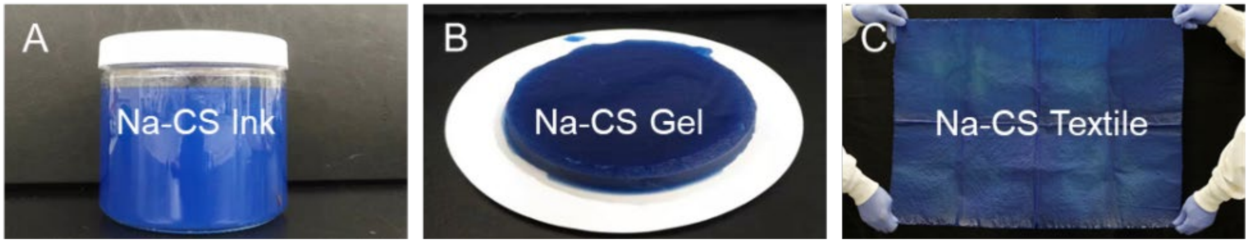

Fig. S21 Other forms of Na-CS from cellulose materials. (A) Na-CS ink. (B) Na-CS gel, and (C) Na-CS cotton textile.

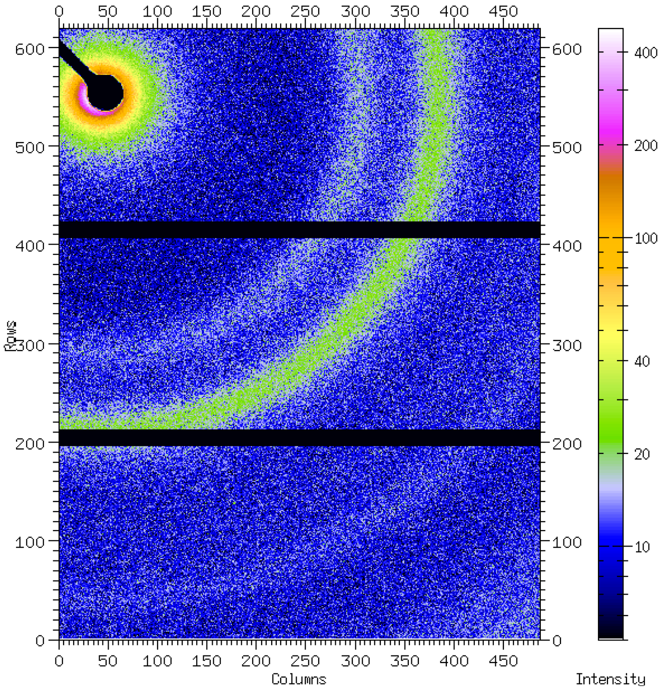

Fig. S22 2D powder XRD pattern of filter paper Na-CS after storing in 20 wt% NaOH solution for more than 2.5 years.

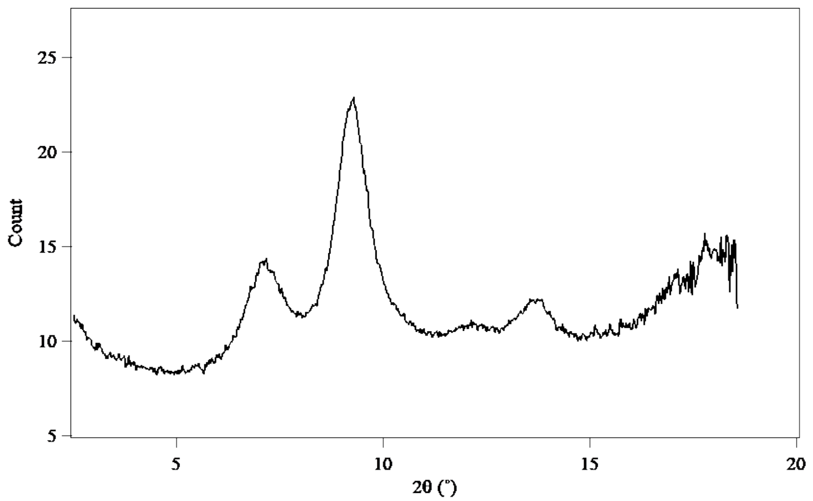

Fig. S23 1D XRD pattern of filter paper Na-CS after storing in 20 wt% NaOH solution for more than 2.5 years. Peaks are consistent with those shown in Fig. S12, indicating no significant degradation after more than 2.5 years.

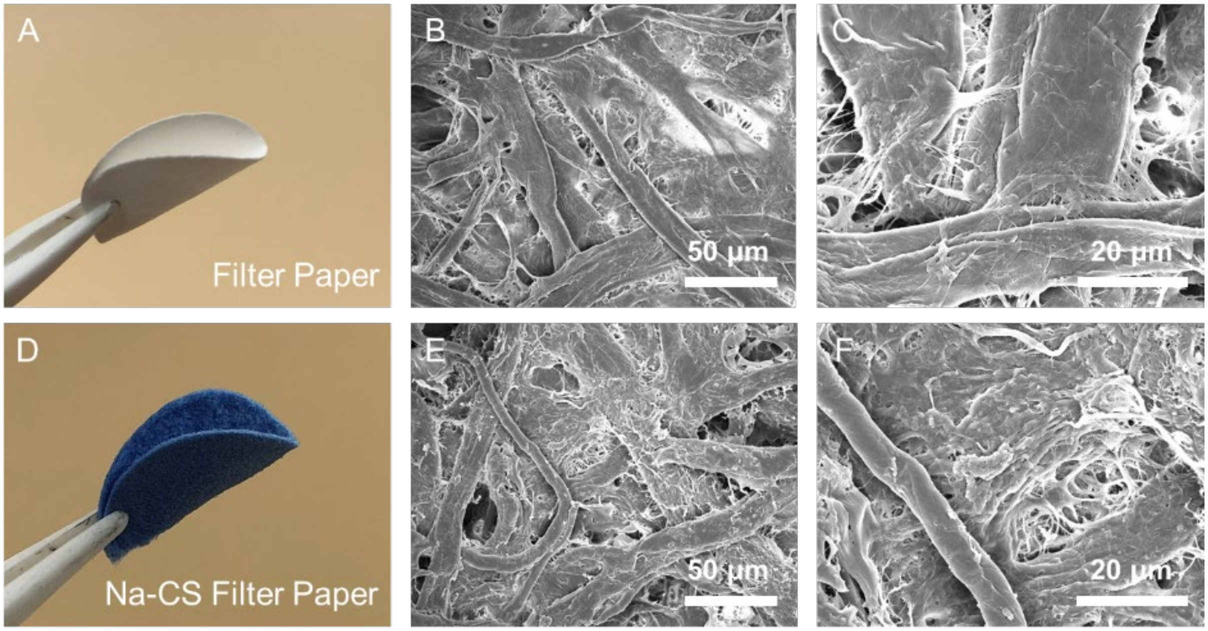

Fig. S24 Filter paper was used as the cellulose starting material for the fabrication of the paper-based Na-CS. (A) Digital photo of the pristine filter paper. (B, C) SEM images of the pristine filter paper at (B) lower and (C) higher magnifications. (D) Digital photo of the Na-CS filter paper. (E, F) SEM images of the Na-CS filter paper at (E) lower and (F) higher magnifications. No discernable difference of the microstructures can be observed between the pristine and Na-CS filter papers.

Data S1 Na Cellulose II_Polymer 1974

Data S2 Na-CS_Model P3221

Data S3 Na-CS_Model P62

REFERENCES AND NOTES

1. D. G. Mackanic, X. Yan, Q. Zhang, N. Matsuhisa, Z. Yu, Y. Jiang, T. Manika, J. Lopez, H. Yan, K. Liu, X. Chen, Y. Cui, Z. Bao, Decoupling of mechanical properties and ionic conductivity in supramolecular lithium ion conductors. *Nat. Commun.* **10**, 5384 (2019).

2. S. Horike, D. Umeyama, S. Kitagawa, Ion conductivity and transport by porous coordination polymers and metal-organic frameworks. *Acc. Chem. Res.* **46**, 2376–2384 (2013).

3. X. Li, H. Zhang, P. Wang, J. Hou, J. Lu, C. D. Easton, X. Zhang, M. R. Hill, A. W. Thornton, J. Z. Liu, B. D. Freeman, A. J. Hill, L. Jiang, H. Wang, Fast and selective fluoride ion conduction in sub-1-nanometer metal-organic framework channels. *Nat. Commun.* **10**, 2490 (2019).

4. Q. Zhang, P.-S. Cao, Y. Cheng, S.-S. Yang, Y.-D. Yin, T.-Y. Lv, Z.-Y. Gu, Nonlinear ion transport through ultrathin metal–organic framework nanosheet. *Adv. Funct. Mater.* **30**, 2004854 (2020).

5. Y.-C. Liu, L.-H. Yeh, M.-J. Zheng, K. C.-W. Wu, Highly selective and high-performance osmotic power generators in subnanochannel membranes enabled by metal-organic frameworks. *Sci. Adv.* **7**, eabe9924 (2021).

6. Z. Man, J. Safaei, Z. Zhang, Y. Wang, D. Zhou, P. Li, X. Zhang, L. Jiang, G. Wang, Serosa-mimetic nanoarchitecture membranes for highly efficient osmotic energy generation. *J. Am. Chem. Soc.* **143**, 16206–16216 (2021).

7. M. Wang, Y. Hou, L. Yu, X. Hou, Anomalies of ionic/molecular transport in nano and sub-nano confinement. *Nano Lett.* **20**, 6937–6946 (2020).

8. H. Deng, S. Grunder, K. E. Cordova, C. Valente, H. Furukawa, M. Hmadeh, F. Gándara, A. C. Whalley, Z. Liu, S. Asahina, H. Kazumori, M. O’Keeffe, O. Terasaki, J. F. Stoddart, O. M. Yaghi, Large-pore apertures in a series of metal-organic frameworks. *Science* **336**, 1018–1023 (2012).

9. H. Zhang, J. Hou, Y. Hu, P. Wang, R. Ou, L. Jiang, J. Z. Liu, B. D. Freeman, A. J. Hill, H. Wang, Ultrafast selective transport of alkali metal ions in metal organic frameworks with subnanometer pores. *Sci. Adv.* **4**, eaaq0066 (2018).

10. S. Han, N. U. H. Alvi, L. Granlöf, H. Granberg, M. Berggren, S. Fabiano, X. Crispin, A Multiparameter pressure-temperature-humidity sensor based on mixed ionic-electronic cellulose aerogels. *Adv. Sci.* **6**, 1802128 (2019).

11. A. Malti, J. Edberg, H. Granberg, Z. U. Khan, J. W. Andreasen, X. Liu, D. Zhao, H. Zhang, Y. Yao, J. W. Brill, I. Engquist, M. Fahlman, L. Wågberg, X. Crispin, M. Berggren, An organic mixed ion-electron conductor for power electronics. *Adv. Sci.* **3**, 1500305 (2016).

12. R. J. Moon, A. Martini, J. Nairn, J. Simonsen, J. Youngblood, Cellulose nanomaterials review: Structure, properties and nanocomposites. *Chem. Soc. Rev.* **40**, 3941–3994 (2011).

13. A. Isogai, Development of completely dispersed cellulose nanofibers. *Proc. Jpn. Acad. Ser. B Phys. Biol. Sci.* **94**, 161–179 (2018).

14. Y. Habibi, L. A. Lucia, O. J. Rojas, Cellulose nanocrystals: Chemistry, self-assembly, and applications. *Chem. Rev.* **110**, 3479–3500 (2010).

15. Y. Ogawa, H. Hidaka, S. Kimura, U.-J. Kim, S. Kuga, M. Wada, Formation and stability of cellulose-copper-NaOH crystalline complex. *Cellulose* **21**, 999–1006 (2014).

16. S. Faucher, N. Aluru, M. Z. Bazant, D. Blankschtein, A. H. Brozena, J. Cumings, J. P. de Souza, M. Elimelech, R. Epsztein, J. T. Fourkas, A. G. Rajan, H. J. Kulik, A. Levy, A. Majumdar, C. Martin, M. McEldrew, R. P. Misra, A. Noy, T. A. Pham, M. Reed, E. Schwegler, Z. Siwy, Y. Wang, M. Strano, Critical knowledge gaps in mass transport through single-digit nanopores: A review and perspective. *J. Phys. Chem. C* **123**, 21309–21326 (2019).

17. J.-M. Lehn, Supramolecular chemistry. *Science* **260**, 1762–1763 (1993).

18. G. F. McLean, T. Niet, S. Prince-Richard, N. Djilali, An assessment of alkaline fuel cell technology. *Int. J. Hydrogen Energ.* **27**, 507–526 (2002).

19. K. Lin, Q. Chen, M. R. Gerhardt, L. Tong, S. B. Kim, L. Eisenach, A. W. Valle, D. Hardee, R. G. Gordon, M. J. Aziz, M. P. Marshak, Alkaline quinone flow battery. *Science* **349**, 1529–1532 (2015).

20. R. MacKinnon, Potassium channels and the atomic basis of selective ion conduction (Nobel lecture). *Angew. Chem.* **43**, 4265–4277 (2004).

21. X. Hou, W. Guo, L. Jiang, Biomimetic smart nanopores and nanochannels. *Chem. Soc. Rev.* **40**, 2385–2401 (2011).

22. J. Shin, J. W. Choi, Opportunities and reality of aqueous rechargeable batteries. *Adv. Energy Mater.* **10**, 2001386 (2020).

23. J. W. Choi, D. Aurbach, Promise and reality of post-lithium-ion batteries with high energy densities. *Nat. Rev. Mater.* **1**, 16013 (2016).

24. M. Pasta, C. D. Wessells, R. A. Huggins, Y. Cui, A high-rate and long cycle life aqueous electrolyte battery for grid-scale energy storage. *Nat. Commun.* **3**, 1149 (2012).

25. M. Wang, H. Meng, D. Wang, Y. Yin, P. Stroeve, Y. Zhang, Z. Sheng, B. Chen, K. Zhan, X. Hou, Dynamic curvature nanochannel-based membrane with anomalous ionic transport behaviors and reversible rectification switch. *Adv. Mater.* **31**, 1805130 (2019).

26. C. Keplinger, J.-Y. Sun, C. C. Foo, P. Rothemund, G. M. Whitesides, Z. Suo, Stretchable, transparent, ionic conductors. *Science* **341**, 984–987 (2013).

27. C. H. Yang, B. Chen, J. J. Lu, J. H. Yang, J. Zhou, Y. M. Chen, Z. Suo, Ionic cable. *Extreme Mech. Lett.* **3**, 59–65 (2015).

28. Y. Li, Q. Fu, S. Yu, M. Yan, L. Berglund, Optically transparent wood from a nanoporous cellulosic template: Combining functional and structural performance. *Biomacromolecules* **17**, 1358–1364 (2016).

29. P. M. Whitaker, I. A. Nieduszynski, E. D. T. Atkins, Structural aspects of sodacellulose II. *Polymer* **15**, 125–127 (1974).

30. T. Okano, A. Sarko, Mercerization of cellulose. II. Alkali–cellulose intermediates and a possible mercerization mechanism. *Appl. Polym.* **30**, 325–332 (1985).

31. C. Prestipino, L. Regli, J. G. Vitillo, F. Bonino, A. Damin, C. Lamberti, A. Zecchina, P. L. Solari, K. O. Kongshaug, S. Bordiga, Local structure of framework Cu(II) in HKUST-1 metallorganic framework: Spectroscopic characterization upon activation and interaction with adsorbates. *Chem. Mater.* **18**, 1337–1346 (2006).

32. G.-F. Chen, Y. Yuan, H. Jiang, S.-Y. Ren, L.-X. Ding, L. Ma, T. Wu, J. Lu, H. Wang, Electrochemical reduction of nitrate to ammonia via direct eight-electron transfer using a copper-molecular solid catalyst. *Nat. Energy* **5**, 605–613 (2020).

33. P. Frank, M. Benfatto, M. Qayyam, B. Hedman, K. O. Hodgson, A high-resolution XAS study of aqueous Cu(II) in liquid and frozen solutions: Pyramidal, polymorphic, and non-centrosymmetric. *J. Chem. Phys.* **142**, 084310 (2015).

34. L. G. Bäck, S. Ali, S. Karlsson, L. Wondraczek, B. Jonson, X-ray and UV-Vis-NIR absorption spectroscopy studies of the Cu(I) and Cu(II) coordination environments in mixed alkali-lime-silicate glasses. *J. Non Cryst. Solids* **3**, 100029 (2019).

35. P. Frank, M. Benfatto, M. Qayyum, $[Cu(aq)]^{2+}$ is structurally plastic and the axially elongated octahedron goes missing. *J. Chem. Phys.* **148**, 204302 (2018).

36. M. Gerloch, The sense of Jahn-Teller distortions in octahedral copper(II) and other transition-metal complexes. *Inorg. Chem.* **20**, 638–640 (1981).

37. C. Yang, Q. Wu, W. Xie, X. Zhang, A. Brozena, J. Zheng, M. N. Garaga, B. H. Ko, Y. Mao, S. He, Y. Gao, P. Wang, M. Tyagi, F. Jiao, R. Briber, P. Albertus, C. Wang, S. Greenbaum, Y.-Y. Hu, A. Isogai, M. Winter, K. Xu, Y. Qi, L. Hu, Copper-coordinated cellulose ion conductors for solid-state batteries. *Nature* **598**, 590–596 (2021).

38. X. Zhang, B. Song, L. Jiang, Driving force of molecular/ionic superfluid formation. *CCS Chem.* **3**, 1258–1266 (2021).

39. I. Vlassiouk, S. Smirnov, Z. Siwy, Ionic selectivity of single nanochannels. *Nano Lett.* **8**, 1978–1985 (2008).

40. S.-J. Chun, E.-S. Choi, E.-H. Lee, J. H. Kim, S.-Y. Lee, S.-Y. Lee, Eco-friendly cellulose nanofiber paper-derived separator membranes featuring tunable nanoporous network channels for lithium-ion batteries. *J. Mater. Chem.* **22**, 16618–16626 (2012).

41. C. Duan, A. Majumdar, Anomalous ion transport in 2-nm hydrophilic nanochannels. *Nat. Nanotechnol.* **5**, 848–852 (2010).

42. R. M. DuChanois, C. J. Porter, C. Violet, R. Verduzco, M. Elimelech, Membrane materials for selective ion separations at the water–energy nexus. *Adv. Mater.* **33**, 2101312 (2021).

43. R. Epsztein, R. M. DuChanois, C. L. Ritt, A. Noy, M. Elimelech, Towards single-species selectivity of membranes with subnanometre pores. *Nat. Nanotechnol.* **15**, 426–436 (2020).

44. H. F. Wang, L. Chen, H. Pang, S. Kaskel, Q. Xu, MOF-derived electrocatalysts for oxygen reduction, oxygen evolution and hydrogen evolution reactions. *Chem. Soc. Rev.* **49**, 1414–1448 (2020).

45. S. Wang, S. S. Park, C. T. Buru, H. Lin, P.-C. Chen, E. W. Roth, O. K. Farha, C. A. Mirkin, Colloidal crystal engineering with metal-organic framework nanoparticles and DNA. *Nat. Commun.* **11**, 2495 (2020).

46. X. Gong, K. Gnanasekaran, Z. Chen, L. Robison, M. C. Wasson, K. C. Bentz, S. M. Cohen, O. K. Farha, N. C. Gianneschi, Insights into the structure and dynamics of metal-organic frameworks via transmission electron microscopy. *J. Am. Chem. Soc.* **142**, 17224–17235 (2020).

47. G. Skorupskii, B. A. Trump, T. W. Kasel, C. M. Brown, C. H. Hendon, M. Dincă, Efficient and tunable one-dimensional charge transport in layered lanthanide metal-organic frameworks. *Nat. Chem.* **12**, 131–136 (2020).

48. J.-H. Dou, M. Q. Arguilla, Y. Luo, J. Li, W. Zhang, L. Sun, J. L. Mancuso, L. Yang, T. Chen, L. R. Parent, G. Skorupskii, N. J. Libretto, C. Sun, M. C. Yang, P. V. Dip, E. J. Brignole, J. T. Miller, J. Kong, C. H. Hendon, J. Sun, M. Dincă, Atomically precise single-crystal structures of electrically conducting 2D metal-organic frameworks. *Nat. Mater.* **20**, 222–228 (2021).

49. A. Anastasopoulou, H. Furukawa, B. R. Barnett, H. Z. H. Jiang, J. R. Long, H. M. Breunig, Technoeconomic analysis of metal-organic frameworks for bulk hydrogen transportation. *Energ. Environ. Sci.* **14**, 1083–1094 (2021).

50. D. DeSantis, J. A. Mason, B. D. James, C. Houchins, J. R. Long, M. Veenstra, Techno-economic analysis of metal-organic frameworks for hydrogen and natural gas storage. *Energy Fuel* **31**, 2024–2032 (2017).

51. M. I. Severino, E. Gkaniatsou, F. Nouar, M. L. Pinto, C. Serre, MOFs industrialization: A complete assessment of production costs. *Faraday Discuss.* **231**, 326–341 (2021).

52. W. L. Teo, W. Zhou, C. Qian, Y. Zhao, Industrializing metal-organic frameworks: Scalable synthetic means and their transformation into functional materials. *Mater. Today*, **47**, 170–186 (2021).

53. Y. Liang, Y. Jing, S. Gheytani, K.-Y. Lee, P. Liu, A. Facchetti, Y. Yao, Universal quinone electrodes for long cycle life aqueous rechargeable batteries. *Nat. Mater.* **16**, 841–848 (2017).

54. D. Zhao, A. Würger, X. Crispin, Ionic thermoelectric materials and devices. *J. Energy Chem.* **61**, 88–103 (2021).

55. A. J. Kropf, J. Katsoudas, S. Chattopadhyay, T. Shibata, E. A. Lang, V. N. Zyryanov, B. Ravel, K. McIvor, K. M. Kemner, K. G. Scheckel, S. R. Bare, J. Terry, S. D. Kelly, B. A. Bunker, C. U. Segre, The new MRCAT (Sector 10) bending magnet beamline at the advanced photon source. *AIP Conf. Proc.* **1234**, 299–302 (2010).

56. M. Newville, IFEFFIT: Interactive XAFS analysis and FEFF fitting. *J. Synchrotron Radiat.* **8**, 322–324 (2001).

57. B. Ravel, M. Newville, ATHENA, ARTEMIS, HEPHAESTUS: Data analysis for X-ray absorption spectroscopy using IFEFFIT. *J. Synchrotron Radiat.* **12**, 537–541 (2005).

58. Y. Jing, Y. Liang, S. Gheytani, Y. Yao, A quinone anode for lithium-ion batteries in mild aqueous electrolytes. *ChemSusChem* **13**, 2250–2255 (2020).

59. S. I. Ajiboye, D. R. Brown, Electron spin resonance study of soluble copper(II)-cellulose complexes. *J. Chem. Soc. Faraday Trans.* **86**, 65–68 (1990).

60. J. Hutter, M. Iannuzzi, F. Schiffmann, J. VandeVondele, CP2K: Atomistic simulations of condensed matter systems. *Wiley Interdiscip. Rev. Comput. Mol. Sci.* **4**, 15–25 (2014).

61. A. Bankura, A. Karmakar, V. Carnevale, A. Chandra, M. L. Klein, Structure, dynamics, and spectral diffusion of water from first-principles molecular dynamics. *J. Phys. Chem. C* **118**, 29401–29411 (2014).

62. S. Grimme, S. Ehrlich, L. Goerigk, Effect of the damping function in dispersion corrected density functional theory. *J. Comput. Chem.* **32**, 1456–1465 (2011).

63. S. Grimme, J. Antony, S. Ehrlich, H. Krieg, A consistent and accurate ab initio parametrization of density functional dispersion correction (DFT-D) for the 94 elements H-Pu. *J. Chem. Phys.* **132**, 154104 (2010).

64. J. VandeVondele, J. Hutter, Gaussian basis sets for accurate calculations on molecular systems in gas and condensed phases. *J. Chem. Phys.* **127**, 114105 (2007).

65. J. VandeVondele, M. Krack, F. Mohamed, M. Parrinello, T. Chassaing, J. Hutter, Quickstep: Fast and accurate density functional calculations using a mixed Gaussian and plane waves approach. *Comput. Phys. Commun.* **167**, 103–128 (2005).

66. J. VandeVondele, J. Hutter, An efficient orbital transformation method for electronic structure calculations. *J. Chem. Phys.* **118**, 4365–4369 (2003).

67. S. Goedecker, M. Teter, J. Hutter, Separable dual-space Gaussian pseudopotentials. *Phys. Rev. B Condens. Matter* **54**, 1703–1710 (1996).

68. G. Bussi, D. Donadio, M. Parrinello, Canonical sampling through velocity rescaling. *J. Chem. Phys.* **126**, 014101 (2007).

69. A. H. Larsen, J. J. Mortensen, J. Blomqvist, I. E. Castelli, R. Christensen, M. Dulak, J. Friis, M. N. Groves, B. Hammer, C. Hargus, E. D. Hermes, P. C. Jennings, P. B. Jensen, J. Kermode, J. R. Kitchin, E. L. Kolsbjerg, J. Kubal, K. Kaasbjerg, S. Lysgaard, J. B. Maronsson, T. Maxson, T. Olsen, L. Pastewka, A. Peterson, C. Rostgaard, J. Schiotz, O. Schutt, M. Strange, K. S. Thygesen, T. Vegge, L. Vilhelmsen, M. Walter, Z. Zeng, K. W. Jacobsen, The atomic simulation environment—A Python library for working with atoms. *J. Condens. Matter Phys.* **29**, 273002 (2017).

70. N. Michaud-Agrawal, E. J. Denning, T. B. Woolf, O. Beckstein, MDAnalysis: A toolkit for the analysis of molecular dynamics simulations. *J. Comput. Chem.* **32**, 2319–2327 (2011).

71. R. Hanson, Jmol—A paradigm shift in crystallographic visualization. *J. Appl. Cryst.* **43**, 1250–1260 (2010).

72. E. S. Gardiner, A. Sarko, Packing analysis of carbohydrates and polysaccharides. 16. The crystal structures of celluloses IVI and IVII. *Can. J. Chem.* **63**, 173–180 (1985).

73. K. Saalwächter, W. Burchard, P. Klüfers, G. Kettenbach, P. Mayer, D. Klemm, S. Dugarmaa, Cellulose solutions in water containing metal complexes. *Macromolecules* **33**, 4094–4107 (2000).

74. O. Hanemann, M. Ballauff, Chain conformation of cellulose in a coordinating solvent. *Macromolecules* **30**, 7638–7640 (1997).

75. W. Burchard, N. Habermann, P. Klüfers, B. Seger, U. Wilhelm, Cellulose in Schweizer's reagent: A stable, polymeric metal complex with high chain stiffness. *Angew. Chemie. Int. Ed.* **33**, 884–887 (1994).

76. A. A. Bagabas, M. Frasconi, J. Iehl, B. Hauser, O. K. Farha, J. T. Hupp, K. J. Hartlieb, Y. Y. Botros, J. F. Stoddart, γ-Cyclodextrin cuprate sandwich-type complexes. *Inorg. Chem.* **52**, 2854–2861 (2013).

77. Y. Nishiyama, J. Sugiyama, H. Chanzy, P. Langan, Crystal structure and hydrogen bonding system in cellulose I_{α} from synchrotron X-ray and neutron fiber diffraction. *J. Am. Chem. Soc.* **125**, 14300–14306 (2003).

78. Y. Nishiyama, P. Langan, H. Chanzy, Crystal structure and hydrogen-bonding system in cellulose I_{β} from synchrotron X-ray and neutron fiber diffraction. *J. Am. Chem. Soc.* **124**, 9074–9082 (2002).

79. P. Langan, Y. Nishiyama, H. Chanzy, X-ray structure of mercerized cellulose II at 1 Å resolution. *Biomacromolecules* **2**, 410–416 (2001).

80. H. Nishimura, T. Okano, A. Sarko, Mercerization of cellulose. 5. Crystal and molecular structure of Na-cellulose I. *Macromolecules* **24**, 759–770 (1991).

81. M. E. Tuckerman, A. Chandra, D. Marx, Structure and dynamics of OH^-(aq). *Acc. Chem. Res.* **39**, 151–158 (2006).

82. M. E. Tuckerman, D. Marx, M. Parrinello, The nature and transport mechanism of hydrated hydroxide ions in aqueous solution. *Nature* **417**, 925–929 (2002).
